# A genetic analysis reveals novel histone residues required for transcriptional reprogramming upon stress

**DOI:** 10.1093/nar/gkaa081

**Published:** 2020-02-17

**Authors:** Cristina Viéitez, Gerard Martínez-Cebrián, Carme Solé, René Böttcher, Clement M Potel, Mikhail M Savitski, Sara Onnebo, Marc Fabregat, Ali Shilatifard, Francesc Posas, Eulàlia de Nadal

**Affiliations:** 1 Cell Signaling Research Group, Departament de Ciències Experimentals i de la Salut, Universitat Pompeu Fabra (UPF), E-08003 Barcelona, Spain; 2 European Molecular Biology Laboratory, Genome Biology Unit, 69117 Heidelberg, Germany; 3 Institute for Research in Biomedicine (IRB Barcelona), The Barcelona Institute of Science and Technology, Baldiri Reixac 10, 08028 Barcelona, Spain; 4 Department of Biochemistry and Molecular Genetics, Feinberg School of Medicine, Northwestern University, IL 60611, USA

## Abstract

Cells have the ability to sense, respond and adapt to environmental fluctuations. Stress causes a massive reorganization of the transcriptional program. Many examples of histone post-translational modifications (PTMs) have been associated with transcriptional activation or repression under steady-state growth conditions. Comparatively less is known about the role of histone PTMs in the cellular adaptive response to stress. Here, we performed high-throughput genetic screenings that provide a novel global map of the histone residues required for transcriptional reprogramming in response to heat and osmotic stress. Of note, we observed that the histone residues needed depend on the type of gene and/or stress, thereby suggesting a ‘personalized’, rather than general, subset of histone requirements for each chromatin context. In addition, we identified a number of new residues that unexpectedly serve to regulate transcription. As a proof of concept, we characterized the function of the histone residues H4-S47 and H4-T30 in response to osmotic and heat stress, respectively. Our results uncover novel roles for the kinases Cla4 and Ste20, yeast homologs of the mammalian PAK2 family, and the Ste11 MAPK as regulators of H4-S47 and H4-T30, respectively. This study provides new insights into the role of histone residues in transcriptional regulation under stress conditions.

## INTRODUCTION

Stress jeopardizes cell viability. In this context, the regulation of gene expression is one of the most important cell responses to achieve rapid adaptation to stress (1,2). Changes in the environment trigger a large common transcriptional response in budding yeast, which is characterized by the re-direction of resources from rapid proliferation to stress protection. Stress-induced genes related to defense against reactive oxygen species and DNA damage, carbohydrate metabolism and energy generation functions are up-regulated, whereas stress-repressed genes, which have growth-related functions, such as translation and ribosome biogenesis, are down-regulated (3–6). Although transcriptome changes have common features, some aspects of the response triggered by specific transcription factors are unique to each individual stress (7–9). Over the past few years, different chromatin modifying and remodeling activities have been dissected in response to stress (e.g. (10–14)). Furthermore, transcriptional stress responses are sophisticated and finely tuned; the magnitude and duration of the response is proportional to the degree of perturbation, and different perturbations result in distinct expression signatures (7,15,16). Several layers of regulatory elements control such dynamic response, from the combinatorial network of transcription factors binding (8,9) to the activity of specific chromatin remodelers, which shape the duration and intensity of the transcriptional response (e.g. SWI/SNF, RSC and SAGA) (11,14,15,17) or attenuate it (INO80 and ISWI) (12,18), according to the cellular requirements.

Chromatin dynamics are essential for transcriptional regulation. Nucleosome dynamics and histone post-translational modifications (PTMs) can influence transcription initiation and elongation by modulating chromatin compaction and accessibility for regulatory proteins (19–22). To date, the most widely described PTMs have been reported to occur in the unstructured histone tails that protrude from the nucleosome core. However, over the last few years, novel modifications have been discovered in the globular domains of histones (23–25), thereby increasing our knowledge of how PTMs respond to and influence transcriptional regulation. High-throughput phenotype analyses of histone residues have been done in various stress conditions and have been instrumental to define histone modification patterns upon stress. For instance, screenings were performed using a comprehensive library of histone H2A and H2B mutants to assess the biological function of each amino acid residue involved in environmental perturbations, including DNA damage and heat stress, as well as to monitor heterochromatin gene silencing and genome stability (26). Also, a library of 486 systematic histone H3 and H4 substitution and deletion mutants was used to probe fitness contributions of each residue to perturbations of chromosome integrity and transcription, mapping global patterns of chemical sensitivities and requirements for transcriptional silencing onto the nucleosome surface (27). In addition, other single gene and genome-wide studies revealed that many histone modifications have far greater effects on the dynamics of gene expression upon a rapid and dramatic reorganization of the yeast transcriptome than on steady-state transcription (e.g. in response to phosphate starvation (28), galactose (29) or diamide (30,31)). Indeed, a database of phenotypes for systematic collections of histone mutants has been reported (HistoneHits), which combines described phenotypes with information about sequences, structures, post-translational modifications, and evolutionary conservation (32). However, to date, high-throughput analysis of osmotic and heat stress-induced transcription on histone mutant libraries has been largely unexplored. Here, we performed high-throughput screenings to identify the histone residues and PTMs required for transcriptional reprogramming upon heat stress and osmostress. This work offers a new comprehensive global map of the histone requirements for transcriptional reprogramming in yeast in response to stress, thus providing further insights into the biology of histone modification. Moreover, following the results of the screening, and as a proof of concept of its effectiveness, we selected the residues H4-S47 and H4-T30 for further analysis. Our results contribute to increasing current understanding of the regulation of these individual histone residues in the context of stress-responsive transcription.

## MATERIALS AND METHODS

### Yeast strains and plasmids

The *Saccharomyces cerevisiae* histone H3/H4 mutant library (27) commercially available from Open Biosystems, and the H2A/H2B SHIMA collection (33) were used for the screenings. A total of 569 histone point mutants were analyzed covering histones H3, H4, H2A and H2B. Specifically, 195 and 150 non-essential point mutants of histones H3 and H4, respectively, were obtained from the Open Biosystems library (27). Of note, for this study we discarded from this library (i) histone mutant residues that are essential for cell viability and carry a histone wild-type copy; (ii) mutants that comprised histone fragment deletions and (iii) multiple amino acid substitutions in the same strain. Additionally, 112 histone mutants of H2A and 112 histone mutants of H2B were obtained from the SHIMA library (34). For the transcriptional screenings, quadruple Venus (qV) fluorescence protein fused to the promoter of stress-responsive genes (p*ALD3-*qV, p*HSP82-*qV and p*STL1-*qV) was used as a reporter. pRS-based reporter plasmids were constructed by cloning stress-induced promoters amplified by PCR from yeast genomic DNA between *SacI* and *SpeI*. Plasmids pSP30 (pRS305-p*ALD3* (-664-0)-Quadruple Venus from (16)), pMT1 (pRS305-p*STL1* (-800-0)-Quadruple Venus and pCV1 (pRS305-p*HSP82* (-800-0)-Quadruple Venus) were used. The plasmids were integrated into wild-type BY4742α (MATα his3Δ1 leu2Δ0 lys2Δ0 ura3Δ0) or Y5563α (*MATα his3Δ*1, *leu2Δ*0, *ura3Δ*0, *met15Δ*0, *can1Δ::*p*MFA1-HIS3*, *lyp1*Δ) strains (35) to construct query strains. Stronger expressing transformants were selected by microscopy.

For H3/H4 query strain construction, BY4742 was transformed with *KasI*-linearized plasmid pRS305 p*ALD3-qV-LEU*, pRS305 p*HSP82-qV-LEU* and pRS305 p*STL1-qV-LEU*, and transformants were plated on SD-*LEU* media to select for the integrated *LEU2* marker and the pRS305 backbone. For H2A/B query strain construction, Y5563 was transformed with the p*MFA1-URA3-NatMX4* cassette to swap *HIS3* by *URA3-NatMX4*, obtaining the Y5563 *can1Δ::*p*MFA1-URA3-NatMX4*. Transformants were selected on YPD plates containing cloNAT. Selected clones were transformed with *KasI*-linearized plasmid pRS305 p*ALD3-qV-LEU*, pRS305 p*HSP82-qV-LEU* and pRS305 p*STL1-qV-LEU*, and transformants were plated on SD-*LEU* media. Finally, *LEU2* marker was swapped by the KanMX4 cassette and transformants were selected on YPD plates containing G418. Additionally, *TRP1* gene was swapped by the HphNT1 cassette and transformants were selected on YPD plates containing hygromycin B. Thus, the following strains were used in this study: YCV1 (BY4742 *MATα leu2::LEU2* p*ALD3-*qV); YSO63 (BY4742 *MATα leu2::LEU2* p*STL1*-qV); YCV4 (BY4742 *MATα leu2::LEU2* p*HSP82*-qV); YCV86 (Y5563 *MATα* p*ALD3-*qV*-KanMX4 can1*Δ::p*MFA1-URA3-NatMX4 TRP1::HPH*); YCV88 (Y5563 MATα p*STL1*-qV-*KanMX4 can1*Δ::p*MFA1*-*URA3-NatMX4 TRP1::HPH*) and YCV87 (Y5563 MATα p*HSP82*-qV-*KanMX4 can1*Δ::p*MFA1*-*URA3-NatMX4 TRP1::HPH*).

### Mating procedure

Manipulations were carried out using a Singer RoTor colony pinning robot. Mating was performed as previously described (35) with some modifications. The histone libraries were arrayed in 96-colony format on YPD plates as were the query strains. Libraries and query strains were combined on YPD plates and incubated at 30°C for one day. The resulting *MATa/α* zygotes were then pinned onto diploid selection media plates (synthetic dextrose medium (SD) lacking uracil and leucine and SD lacking histidine and containing geneticine (G418) (200 mg/l) for H3/H4 and H2A/B, respectively) and incubated for one day at 30°C.

Selected diploid cells were then transferred into sporulation medium (2% agar, 1% potassium acetate, supplemented with leucine and tryptophan, lacking uracil or histidine for H3/H4 and H2A/B, respectively) and incubated for 6 days at 22°C.

The *MATa* spore progeny was selected using various media. The media used for the selection of H3/H4 *(MATa URA3 LEU2 NatR)* progeny was: monosodium glutamate (MSG) lacking histidine, arginine, and uracil, and containing canavanine (50 mg/l) and nourseothricin (cloNAT) (200 mg/l) for the first selection round and MSG lacking histidine, arginine, uracil, and leucine, and containing canavanine (50 mg/l) and cloNAT (200 mg/l) for the second and third selection rounds. The media used for the selection of H2A/H2B (*MATa HIS3 LEU2 TRP1 kanR)* progeny was: MSG lacking histidine, arginine, uracil, leucine, and tryptophan and containing canavanine (50 mg/l) and cloNAT (200 mg/l) for the first selection round, and MSG lacking histidine, arginine, uracil, leucine, and tryptophan and containing canavanine (50 mg/l), cloNAT (200 mg/l) and G418 (200 mg/l) for the second and third selection round. Each selection round involved two days of incubation at 30°C.

The final genotype of the new libraries was: *MATa his3Δ200 leu2Δ0 lys2Δ0 trp1Δ63 ura3Δ0 met15Δ0 can1::*pMFA1-HIS3 hht1-hhf1::NatMX4 hht2-hhf2::[HHTS-HHFS]*-URA3 p-X-qV-LEU2 for H3/H4 and MATa (hta1-htb1)::LEU2, (hta2-htb2)::TRP1, his3Δ200 leu2Δ1 ura3-52 trp1Δ63 lys2-128Δ [HTA1-HTB1]*-HIS3 can1::pMFA1-URA3-NatMX4 p-X-qV-KanMX4 for H2A/H2B. * indicates either wild-type or mutant allele of the corresponding histone.

### Single-cell transcriptional screenings

Yeast strains were grown on 96-well plates (200 μl/well) and incubated at 25°C at all steps during the screenings. At least, three biological replicates of each yeast strain were grown to log phase and subjected to heat stress (39°C) or osmostress (0.4 M NaCl) for 45 min. Protein translation was stopped by the addition of cycloheximide (CHX) (0.1 mg/ml), and then cells were incubated in the dark for 1 h. The fluorescence of 10 000 cells per well was measured by flow cytometry (FACScanto™) and after cell gating only wells with at least 4000 single cells were considered for analysis. Stress-induced expression was quantified using the fluorescence median value of the stressed wild-type strain as 100% reference. The raw data (percentage of the mutant compared to the wild type strain) of all the available biological replicates for each histone mutant and promoter is shown in Supplementary Table S1. Mutants showing at least a 15% increase or decrease compared to the wild-type strain were considered up-regulated (UP) or down-regulated (DOWN), respectively. No differences (NO) were considered when mutant average was between 85 and 115% or less than two replicates were affected. When the histone mutant basal expression was 50% higher or lower than wild-type cells, the transcriptional effect was classified as ‘BASAL’. Any transcriptional effect under stress was not considered when a basal effect was present. Data were analyzed using Flowjo software.

### 3D analysis

Tridimensional representations were done using Pymol (The PyMOL Molecular Graphics System, Version 2.3.3 Schrödinger, LLC) and the yeast nucleosome crystal structure, whose accession number in the Protein Data Bank is 1ID3 (36).

### Northern blot

Histone collection mutants, BY4741 *STE11*Δ strain or strains harboring the inducible expression system ADGEV (37) to promote the expression of a constitutively activated version of Cla4 (pRS426 p*GAL1*-*CLA4 ^Δ^^N^* from amino acids 496–842) by the addition of 100 nM β-estradiol (Sigma-Aldrich, St Louis, MO, USA) for 2 h, as described in (38), were grown to log phase on YPD medium and then subjected to osmostress (0.4 M NaCl) or heat stress (39°C) for the indicated times. Total RNA was extracted, resolved in 1% agarose gels and transferred to nylon membranes. Expression of specific genes was probed using radiolabeled PCR fragments (High Prime DNA Labeling Kit, Sigma-Aldrich) containing a region of the open reading frame of *ALD3* (0.6 kb), *HSP12* (0.3 kb), *CLA4* (0.75 kb), *CTT1* (1.7 kb), *HXT5* (0.65 kb), *DDR2* (0.16 kb), *ADH1* (1.05 kb), *STE11* (1.1 kb), *LEXA* (0.6 kb), *RDN18* (1 kb) and *ENO1* (1.3 kb). Signals were quantified with a Fujifilm BAS-5000 phosphorimager and ImageQuantTLsoftware. Specifically, quantifications were done by normalizing by the loading control gene and then in respect to the maximum intensity point. Images from a representative experiment are shown. The mean ± SD data of at least three separate experiments and images from a representative experiment are shown.

### RNA-sequencing and analysis

Yeast strains were cultured to mid-log phase in rich medium and then subjected or not to osmostress (15 min 0.4 M NaCl) for H4-S47 wild-type and H4-S47D strains or heat stress (10 min 39°C) for H4-T30 wild-type, H4-T30A and H4-T30D strains. Three biological replicates for each condition were performed. Total RNA was extracted by using the standard hot phenol and glass-bead protocol. Libraries were prepared using the TruSeq stranded mRNA sample preparation kit v2 (Illumina) following the manufacturer's protocol, starting with 1 μg of total RNA for poly(A)-mRNA selection. Libraries were first analyzed on an Agilent Bioanalyzer using a DNA 1000 chip to check the size distribution. Libraries were then quantified by qPCR using the KAPA kit (KapaBiosystems) prior to sequencing with 50 bp single-end reads on an Illumina HiSeq 2500 with v4 sequencing chemistry. Quality control of raw sequencing reads was performed by FASTQC; all samples passed the imposed quality restrictions and were used for further analysis. RNA-seq reads were mapped to the sacCer3 reference genome (obtained via UCSC goldenPath) using TopHat2 v2.1.0 (35) with default parameters. Quantification was performed by featureCounts v1.5.1 (36) using the Ensembl R64-1-1 annotation and only considering uniquely mapping reads. Subsequent analyses were conducted using the statistical programming language R, and the DESeq2 package (v.1.14.1; (37)) was used for library size normalization (RLE) and differential expression testing. To obtain stringent genes sets, we only defined genes that significantly (FDR < 0.05) exceeded an absolute log_2_-fold change (log_2_FC) of 1 as responsive to osmotic or heat stress, respectively (function results(), parameters: (altHypothesis = ‘greaterAbs’, lfcThreshold = 1)). In contrast, to be able to thoroughly capture the effect of the histone mutants in basal conditions, we did not apply such a restriction (log_2_FC ≠ 0, FDR < 0.05). Lastly, the effect of histone mutants on the transcriptional stress response was investigated using an interaction term (∼condition + genotype + condition:genotype) without restrictions regarding the minimum log_2_FC (log_2_FC ≠ 0, FDR < 0.05). Results were visualized using the ggplot2 R-package (http://ggplot2.org). Functional enrichment analyses for histone mutant-affected genes were performed using the gProfileR R-package (39), using all corresponding stress-responsive genes as background to rule out any biased enrichment for stress-specific terms. To define Cla4, Ste11 and Ste20 targets, we utilized published microarray experiments for kinase knock-out mutants (40) and kept all genes with *P* < 0.05 and an absolute *M*-value exceeding |*M*| > 0.5 before employing Fisher's exact test to identify significant enrichments of kinase targets among the affected genes of each histone mutant. RNA-seq data have been deposited in the Gene Expression Omnibus (GEO) database (GSE130549). All RNA-seq experiments were run in triplicates.

### Spot assay

Wild-type and the indicated histone mutant strains were grown to mid-log exponential phase in YPD, and serial dilutions (2-fold) were spotted on agar YPD plates according to each experiment (YPD, 1.2 M NaCl). Cells were allowed to grow at 30°C (osmotic stress) or at 39°C (heat stress) for 3–5 days. Images from a representative experiment are shown.

### Growth curves

Strains were inoculated into a final volume of 200 μl of YPD with or without 1.2 M NaCl in 96-well plates (initial OD_660_ 0.025). Specific wells were inoculated with medium only for background correction purposes. Cells were grown at 30°C or 39°C in a thermostated microplate reader (Synergy H1 Multi-Mode Reader, BioTek) with double orbital shaking. Optical density (OD) was measured at 660 nm for 20–40 h. Data are the means ± SD of a minimum of three technical replicates from a representative experiment.

### Design of recombinant histone peptides

Short histone H4 peptides were designed to contain amino acids S47 or T30 wild type (wt) or the alanine mutant (Ser/Thr to Ala). For H4-S47 experiments, the sequence NH_2_-ARRGGVKRI[S/A]GLIYEEVRAV-COOH covers amino acid residues from 38 to 57 and for H4-T30 experiments, the sequence NH_2_-GRGKGGKGLGKGGAKRHRKILRDNIQGI [T/A] KPAIRRLARRGGVKRI-COOH covers amino acid residues from 2 to 46. PCR fragment (H4-T30) or oligonucleotides (H4-S47) with the corresponding wild-type or alanine residue were cloned into pGEX6P1 vector to obtain recombinant GST-fused peptides.

### Expression and purification of recombinant histone peptides

Recombinant GST-proteins were expressed in *E. coli* BL21 strain first grown at 37°C to OD_600_ = 0.4 and finally induced by the addition of 1 mM isopropylthiogalactoside (IPTG) for 6 h at 25°C. Cells were collected by centrifugation, lysed in buffer STET (10 mM Tris pH 8.0, 100 mM NaCl, 1 mM EDTA pH 8.0, 5% Triton X-100, 2 mM dithiothreitol (DTT), 1 mM phenylmethylsulfonyl fluoride (PMSF), 1 mM benzamidine, 2 μg/ml leupeptin and 2 μg/ml pepstatin), and sonicated. The extract was cleared by high speed centrifugation (7000 *g*). GST-histone peptides were pulled down from the supernatant using glutathione-sepharose beads (GE Healthcare) for 2 h at 4°C. Beads were extensively washed. Finally, bound proteins were eluted in 10 mM reduced glutathione (GSH), 50 mM Tris–HCl pH 9.5 and 2 mM DTT.

### Kinase purification

Kinase-tagged strains from the yeast TAP collection (41) were grown to mid-log phase at 30°C in 50 ml of YPD and collected by brief centrifugation at 4°C. The BY4741 *HOG1*Δ *STE7*Δ strain bearing the plasmid with the constitutively activated version of Ste11 (Ste11^ΔN^ from amino acids 365 to 717) (42) pGAG-*STE11^Δ^^N^* (PGAL1-GST, URA3, 2μ) was grown to mid-log phase in SD medium-URA, and protein expression was induced by switching the growth medium to YPGal 2% for 6 h. Proteins were extracted in buffer A (20 mM Tris–HCl, pH 8, 150 mM NaCl, 15 mm EDTA, 15 mm EGTA, 2 mm DTT, 0.1% Triton X-100, 1 mM PMSF, 1 mM benzamidine, 2 μg/ml leupeptin, 2 μg/ml pepstatin, 10 mM sodium fluoride, 25 mM β-glycerophosphate, 1 mM sodium orthovanadate and 1 mM sodium pyrophosphate), and the cell wall was disrupted mechanically with glass beads. The extract was cleared by centrifugation, and the supernatant was incubated with rabbit IgG-agarose beads (Sigma) for TAP-tagged kinases or with glutathione-sepharose beads (GE Healthcare) for GST-tagged Ste11^ΔN^, for 2 h at 4°C. After washing, beads bound to TAP-tagged kinases or eluted GST-tagged Ste11^ΔN^ protein were used without freezing for *in vitro* kinase assays.

### *In vitro* kinase assay

After purification, kinases were pre-activated with kinase buffer (50 mM Tris–HCl pH 7.5, 10 mM MgCl2, 2 mM DTT and 50 μM ATP) for 5 min at 30°C. Next, 1 μg of the purified full-length H4 histone (commercialized by Histone Source REF 00152), the full-length H4-S47A and H4-T30A custom made by Histone Source, or the short peptides was added to the kinase-bound bead mixture, together with radiolabeled γ-^32^P ATP (0.1 μCi/μl), and incubated for 30 min at 30°C. The reaction was stopped by the addition of 5× SDS loading buffer. Labeled proteins were resolved by SDS/PAGE, transferred to a nylon membrane and detected by autoradiography. GST-fused proteins and tagged kinases were detected by western blot using anti-GST anti-PAP (Sigma Aldrich, cat n° 27457701 and P1291, respectively). Full-length histones and kinase Ste11^ΔN^ were visualized with Naphthol Blue (Sigma Aldrich, cat n°N3393).

### Phosphoproteomics

Cell culture and lysis: wild-type cells were grown overnight at 30°C in YPAD and diluted to OD 0.1 in 250 ml fresh YPAD media. Cultures were collected by centrifugation at 4000 *g* for 15 min when OD_600_ reached ∼0.7 and immediately frozen in liquid nitrogen. Pellets were washed with PBS and cells were lysed using Lysis buffer A (20 mM Tris pH 8.0, 15 mM EDTA pH 8, 15 mM EGTA pH 8.0, phoStop phosphatase inhibitors and 0.1% Triton X-100) and glass beads beating at 4°C for 10 min. Sodium deoxycholate (SDC), Triton X-100, urea, Tris(2-carboxyethyl)phosphine (TCEP) and chloroacetamide (CAA) were added to final concentrations of 1%, 1%, 6 M, 5 mM and 30 mM, respectively. MgCl_2_ was added to a final concentration of 40 mM before addition of benzonase (Merck Millipore, 1% final concentration) and sonication using a bioruptor XL (Diagenode, 45 cycles of 30 s on/30 s off). Methanol/chloroform protein precipitation was then performed and the precipitate was resuspended in the digestion buffer (100 mM HEPES pH 8.5, 1% SDC, 5 mM TCEP and 30 mM CAA) before addition of trypsin at a 1:25 ratio (w/w). Digestion was performed overnight at room temperature. Then, samples were acidified with trifluoroacetic (TFA, final concentration 1%) and centrifuged at 14 000 rpm for 5 min to pellet SDC precipitate. The supernatant was loaded onto a Waters t-C18 SePak 200 mg column and peptides were washed twice with 1 ml 0.1% TFA before elution with 500 μl of 40% acetonitrile acidified with 0.1% TFA, followed by lyophilisation.

*Phosphopeptide enrichment*: The phosphopeptide enrichment was performed as previously described (43), with few modifications. Lyophilized peptides were resuspended in 70% acetonitrile/0.07% TFA (buffer A) before injection on a ProPac IMAC-10 column (Thermo Fisher Scientific, 4 × 50 mm) previously loaded with Fe^3+^ cations. Peptides were loaded for 6 min at a flow rate of 400 μl/min using an Ultimate 3000 UHPLC liquid chromatography system (Thermo Fisher Scientific). Peptides were then washed with 100% buffer A for 6 min at 1 ml/min, before elution by switching to 50% buffer B (0.3% ammonia) for 2 min, at a flow rate of 500 μl/min. The fraction containing phosphopeptides was collected and lyophilized.

High pH fractionation: Phosphopeptides high pH fractionation was performed using in-house packed C18 microcolumns. To do so, gel loader tips were plugged with C18 resin (Affinisep AttractSPE C18 disks) and packed with 1 mg of C18 material (Dr Maisch, 5 μm, 120 Å). Lyophilized phosphopeptides were resuspended in 40 μl 20 mM ammonium formate at pH 10 (buffer A), loaded onto the microcolumn by centrifugation (with a loading speed ≈ 10 μl/min) then washed with 10 μl buffer A. Phosphopeptides were fractionated using a gradient of acetonitrile consisting of sequential elutions with 10 μl of the following solutions: 1%, 3%, 5%, 7%, 9%, 11%, 13%, 15%, 17%, 19%, 21%, 23%, 24%, 26%, 28%, 30%, 40% acetonitrile/buffer A (with an elution speed ≈ 10 μl/min). The flow-through and wash were collected and pooled, while the other elutions were pooled as follow: F1 = 1%, 13%, 24%; F2 = 3%, 15%, 26%; F3 = 5%, 17%, 28%; F4 = 7%, 19%, 30%; F5 = 9%, 21%, 40%; F6 = 11%, 23%.

LC–MS/MS measurements: LC–MS/MS experiments were performed using an UltiMate 3000 RSLCnano system (Thermo Fisher Scientific) coupled to a Fusion Lumos Tribrid mass spectrometer (Thermo Fisher Scientific). Phosphopeptides were resuspended in a mixture of 50 mM citric acid and 1% formic acid, first trapped on a cartridge for 3 min at a flow rate of 30 μl/min (Precolumn; C18 PepMap 100, 5 μm, 300 μm i.d. × 5 mm, 100 Å) before separation on an analytical column (Waters nanoEase HSS C18 T3, 75 μm × 25 cm, 1.8 μm, 100 Å) using a linear gradient from 8% to 25% solvent B (0.1% formic acid in LC‐MS grade acetonitrile), followed by an increase to 80% solvent B and column re-equilibration with 100% solvent A (0.1% formic acid in LC–MS grade water, total analysis time of 90 min).

The mass spectrometer was operated in positive ion mode with a spray voltage of 2.4 kV and a capillary temperature of 275°C. Full-scan MS1 spectra were acquired in the Orbitrap with a scan range of 375–1500 *m*/*z* at a resolution of 120 000 (maximum injection time of 50 ms and automatic gain control (AGC) set to 4e5 charges). The mass spectrometer was operated in data dependent acquisition mode and the 15 most intense precursors with charge states 2–6 and a minimum intensity of 2e5 were sequentially isolated (isolation window of 1.6 *m*/*z*) and fragmented by higher-energy collisional dissociation (HCD, normalized collision energy of 32%) with a 16 s dynamic exclusion window. MS/MS spectra were acquired at a resolution of 30 000 in the Orbitrap with a maximum injection time of 110 ms and an AGC target of 1e5 charges.

Peptide and protein identification: The raw data files were processed by the MaxQuant software (44) (version 1.6.2.3) and searched against a reviewed *S. cerevisiae* database (UniProt, April 2019), with the following parameters: trypsin digestion (cleavage at the C-term of lysine and arginine, even when followed by a proline residue) with a maximum of three missed cleavages, fixed carbamidomethylation of cysteine residues, variable oxidation of methionine residues, variable acetylation of protein N-terminus, as well as variable phosphorylation of serine, threonine and tyrosine residues. Mass tolerance was set to 4.5 ppm at the MS1 level and 20 ppm at the MS2 level. A score cut-off of 40 was used for modified peptides, the false discovery rate was set to 0.01 and the minimum peptide length to seven residues. Results are shown in Supplementary Table S2.

Annotated spectra corresponding to the H4-T30 and H4-S47 phosphorylation events was performed by the Interactive Peptide Spectral Annotator software (IPSA) (45), with a 20 ppm mass tolerance. The mass spectrometry proteomics data have been deposited to the ProteomeXchange Consortium via the PRIDE (46) partner repository with the dataset identifier PXD017134.

### Chromatin immunoprecipitation (ChIP) assays

Yeast strains with either Cla4 or Ste20 tagged with HA plus the deletion of the endogenous *SHO1*, *STE11* or *HOG1* genes were grown to early log phase (OD_660_ 0.4–0.6). Then 50-ml samples were exposed to 0.4 M NaCl for the indicated length of time. For crosslinking, cells were treated with 1% formaldehyde for 20 min at room temperature. Glycine was added to a final concentration of 125 mM for 15 min. Cells were collected, washed four times with cold TBS (20 mM Tris–HCl, pH 7.5, and 150 mM NaCl), and kept at –20 °C for further processing. Cell pellets were resuspended in 0.3 ml cold lysis buffer (50 mM HEPES–KOH, pH 7.5, 140 mM NaCl, 1 mM EDTA, 0.1% sodium deoxycholate, 1% Triton-X 100, 1 mM PMSF, 2 mM benzamidine, 2 μg/ml leupeptin, 2 μg/ml pepstatin, and 2 μg/ml aprotinin). An equal volume of glass beads was added, and cells were disrupted by vortexing (with Vortex Genie) for 13 min on ice. The beads were then discarded, and the crosslinked chromatin was sonicated with water bath sonicator (Bioruptor) to yield an average DNA fragment size of 350 bp (range, 100–850 bp). Finally, the sample was clarified by centrifugation at 16 000 *g* for 5 min at 4°C. Supernatants were incubated with 50 μl anti-HA 12CA5 monoclonal antibody (hybridoma supernatant) precoupled to pan mouse IgG DynabeadsTM (Invitrogen, cat no. 11042). After 2 h at 4°C under constant rotation, the beads were washed twice for 4 min in 1 ml lysis buffer, twice in 1 ml lysis buffer with 500 mM NaCl, twice in 1 ml washing buffer (10 mM Tris-HCl pH 8.0, 0.25 M LiCl, 1 mM EDTA, 0.5% N-P40 and 0.5% sodium deoxycholate) and once in 1 ml TE (10 mM Tris–HCl pH 8.0, and 1 mM EDTA). Immunoprecipitated material was eluted twice from the beads by heating for 10 min at 65 °C in 50 μl elution buffer (25 mM Tris–HCl pH 7.5, 10 mM EDTA and 0.5% SDS). To reverse crosslinking, samples were adjusted to 0.3 ml with elution buffer and incubated overnight at 65 °C. Proteins were digested by adding 0.5 mg/ml Proteinase K (Novagen, 71049) for 1.5 h at 37°C. DNA was then extracted with phenol–chloroform–isoamyl alcohol (25:24:1) and chloroform. It was finally precipitated with 48% (v/v) of isopropanol and 90 mM NaCl for 2 h at −20 °C and resuspended in 30 μl of TE buffer. Quantitative PCR analysis of the corresponding promoter regions was performed. Locations are indicated by the distance from the respective ATG initiation codon: *ALD3* promoter (–570/–248); *HSP12* promoter (–304/–107); operons LexA at -400 bp of *ADH1* promoter; and *TEL* (telomeric region on the right arm of chromosome VI) for background subtracting purposes. Experiments were done on at least three independent chromatin preparations, and quantitative PCR analysis was performed in real time with Power SYBR Green PCR Master Mix (Applied Biosystems) using an Applied Biosystems 7.700 sequence detector. Immunoprecipitation efficiency was calculated in triplicate by normalizing the amount of PCR product in the immunoprecipitated sample by that in the TEL sequence control. The binding data are presented as fold induction against the non-treated wild-type strain. Data represent the mean ± SD of at least three separate experiments.

### Kinase-LexA tethering

The sequences of the protein LexA followed by a single HA tag and *CLA4^Δ^^N^*or *STE11^Δ^^N^* were introduced under the control of *GAL1* promoter in a pRS426 vector. Empty LexA-HA construct was used as control. Vectors were transformed in BY4741 strains harboring the inducible expression system ADGEV (37) and the sequence of 8 LexA operons integrated at –400 bp of *ADH1* coding region. Transformants were grown to mid log phase on YPD medium and expression of the fused protein LexA-HA, LexA-HA-*CLA4^Δ^^N^* or LexA-HA-*STE11^Δ^^N^* was induced with 100 nM β-estradiol (Sigma-Aldrich, St Louis, MO, USA) for the indicated time-points. Samples were prepared for northern blot and ChIP analysis as described above.

### Statistical analysis

Data are reported as mean ± SD. If not declared otherwise, statistical significance was assessed using a Student's *t* test for equality of means, two-tailed and equal variance assumed. *P* < 0.05 was considered significant. *P < 0.05, **P < 0.01.

## RESULTS

### High-throughput analysis of transcriptional response of histone mutants to stress

**Figure 1.**
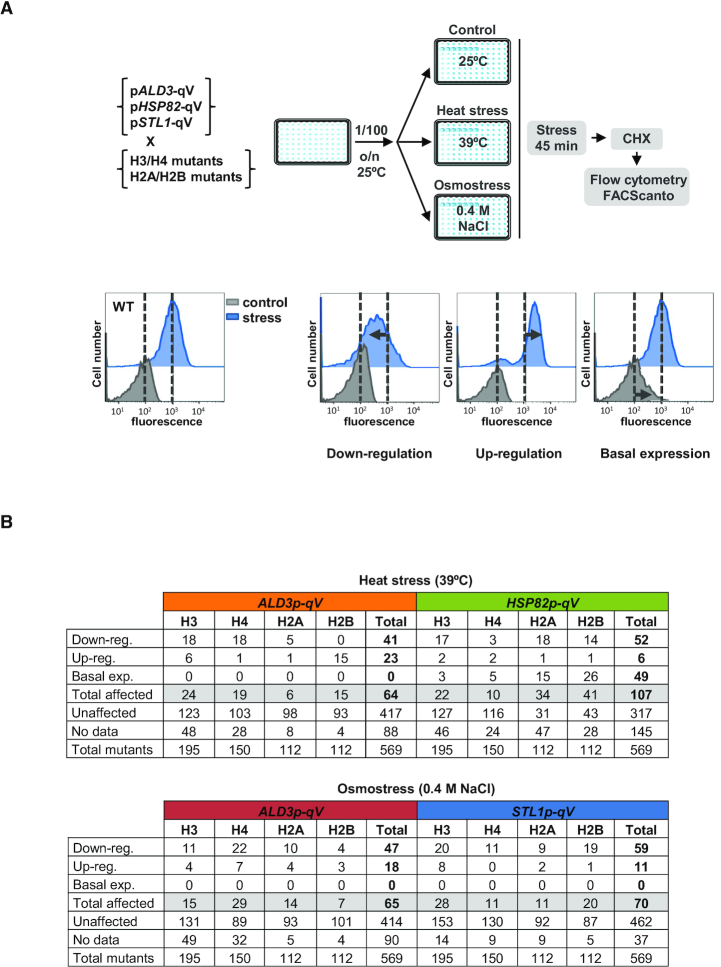
High-throughput screenings of histone residues required for transcriptional regulation upon stress. (**A**) Experimental strategy of the genetic screenings. Yeast cells were diluted using an automated robot (RoTor^®^) and grown at 25°C to mid-log phase; cells were then subjected to heat (39°C) or osmotic (0.4 M NaCl) stress for 45 min. Cycloheximide (CHX) was added and cells were incubated for 1 h in dark and 10 000 cells per condition were measured by flow cytometry using a 96-well plate reader (FACScanto™). Results were analyzed using Flowjo software; dotted black lines represent the level of transcription of the wild-type (WT) strain in control (gray) and stress (blue) conditions. Mutants showing a transcriptional defect were classified into three categories: down-regulation (lower transcription than the stressed WT), up-regulation (higher transcription than the stressed WT) and basal expression (transcriptional defect in the absence of stress in comparison to WT). (**B**) Table summarizing the transcriptional defects of the histone mutants in response to heat- and osmo-stress. For a detailed list see Supplementary Table S1.

In response to environmental fluctuations, yeast cells induce a rapid and massive transcription of stress-responsive genes and down-regulation of growth-related genes (3,7). Since histones play a key role in transcription-related processes, we sought to determine the histone residues essential for transcriptional reprogramming upon stress. To this end, by means of high-throughput screenings, we measured transcription initiation in response to stress at single cell level for 569 histone point mutants. We used two histone mutant libraries that covers the fours histones H3, H4, H2A and H2B: a histone library, which contains 195 and 150 non-essential point mutants of histones H3 and H4, respectively (Open Biosystems) (27) and the SHIMA library with 224 H2A and H2B histone point mutants (34). To quantify the transcriptional output induced by stress, we used a reporter system based on quadruple Venus (qV) fluorescent protein expressed under the control of three specific stress-responsive promoters dependent on different transcription factors: *ALD3*, induced in response to heat and osmostress and regulated by Msn2/4 transcription factors; *HSP82*, induced in response to heat stress and controlled by Hsf1; and *STL1*, induced in response to osmostress and regulated by Hot1 and Sko1 (8,9,47). High-throughput mating of the query strains containing these reporter genes with the aforementioned histone libraries allowed us to generate new libraries harboring both the histone point mutation and the reporter gene (see Methods). Next, we used flow cytometry to analyze the expression of p*ALD3*-qV after exposure to heat shock (39°C) and osmostress (0.4 M NaCl), p*HSP82*-qV upon heat stress, and p*STL1*-qV upon osmostress, comparing the fold induction in wild-type and mutant strains. Additionally, transcriptional defects in the absence of stress were also identified (schema shown in Figure 1A).

**Figure 2.**
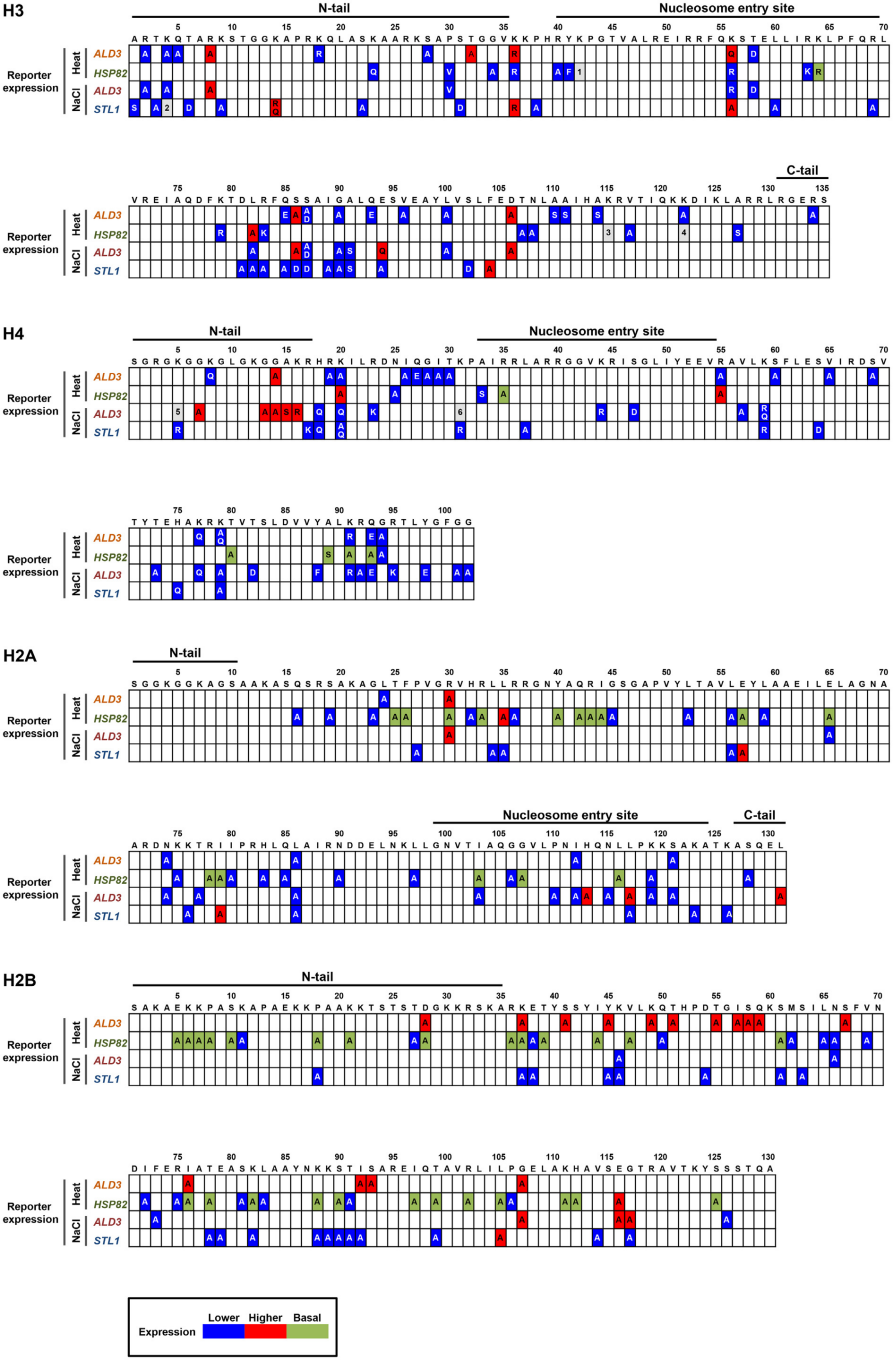
Comprehensive map of the histone residues required for transcriptional response upon stress. The results from the transcriptional screenings are shown in the primary structure of each core histone for each reporter and each stress condition. Transcriptional defects upon stress in the histone mutants (indicated by the single-letter amino acid code) compared to the wild-type strain are shown in blue (expression down-regulated), red (expression up-regulated) or green (higher expression in basal conditions) boxes. Gray boxes: 1: up-regulated (mutant A) and down-regulated expression (mutant R). 2: up-regulated expression (mutants A, R and Q). 3: higher expression in basal conditions (mutant A) and down-regulated expression (mutant R). 4: higher expression in basal conditions (mutant R) and down-regulated expression (mutant Q). 5: up-regulated (mutant A) and down-regulated expression (mutant Q). 6: up-regulated (mutant A) and down-regulated expression (mutant R). For a detailed list of all mutant assayed and their outcomes see Supplementary Table S1.

We identified 47 and 41 histone mutants required to activate p*ALD3*-qV in response to heat and osmotic stress, respectively. p*HSP82*-qV expression was impaired upon heat stress in 52 mutants and p*STL1*-qV was down-regulated upon osmotic stress in 59 mutants. The number of histone mutants that showed up-regulation of the transcriptional response to both stresses was significantly lower (23 in heat- and 18 in osmo- histone mutants in p*ALD3*-qV, 6 in p*HSP82*-qV and 11 in p*STL1*-qV). Moreover, in the absence of stress, the basal expression of only p*HSP82*-qV was up-regulated in 49 cases (Figure 1B and Supplementary Table S1). To obtain a global picture of the histone residues involved in the transcriptional regulation of stress-responsive genes upon stress, we represented all the data obtained from the screenings in the primary structure of the four core histones (Figure 2). When comparing the expression of endogenous stress-dependent genes (*ALD3*, *HSP82* or *STL1*) to the reporter genes, we found similar transcriptional defects in a high percentage of cases, which encouraged us to proceed with our analyses (see two specific examples below). Along the same lines, we also observed that mutations on residues previously described to regulate transcription appeared in our screenings (see discussion).

### Histone residues required for transcription upon stress are specific for each stress condition and promoter, potentially modifiable and located on the nucleosome surface

**Figure 3.**
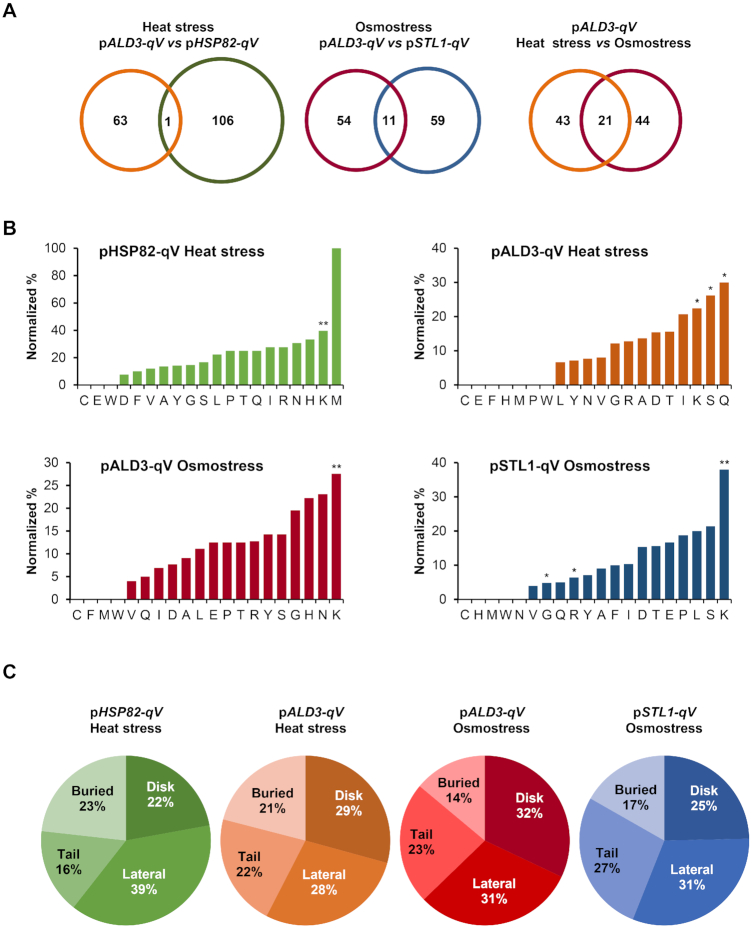
Histone residues required for transcription upon stress are specific for each stress condition and promoter, potentially modifiable and located on the nucleosome surface. (**A**) Venn diagrams showing the overlap between p*ALD3-qV* (orange) and p*HSP82-qV* (green) in response to heat stress, p*ALD3-qV* (red) and p*STL1-qV* (blue) in response to osmostress, and p*ALD3-qV* in response to heat- (orange) and osmo- (red) stresses. (**B**) Percentage of altered amino acids normalized by the total histone amino acid abundance for the indicated conditions. An hypergeometric test was performed to score the significance of the enrichments (*P < 0.05, **P < 0.01) (**C**) Spatial distribution of the histone mutants identified in the transcriptional screenings on the nucleosome structure normalized by the total histone amino acid abundance per category. Histone residues can be located on the unstructured tails (tail), inside the histone structure (buried), and on the histone surface that contacts DNA (lateral) or on the histone surface that does not contact DNA (disk).

To assess promoter specificity, we compared the histone mutants causing transcriptional defects in p*ALD3-qV* and p*HSP82-qV* reporters (promoters governed by the unrelated Msn2/4 and Hsf1 transcription factors respectively) in response to a similar stress. Upon heat stress, only one histone mutant (H4-G94A) exhibited the same phenotype in both reporters (Figure 3A; left Venn diagram). A similar pattern emerged when we analyzed the overlap upon osmostress, where only eleven mutants induced the same transcriptional defects in p*ALD3-qV* and p*STL1-qV* reporters (both promoters also governed by distinct transcription factors) (H3-L82A, H3-S87D, H3-G90A, H3-A91S, H4-H18Q, H4-K20Q, H4-K31R, H4-K59R, H4-K79A, H2A-L86A and H2B-K46A) (Figure 3A; middle Venn diagram). When different cellular stresses (heat *versus* osmotic stress) were compared, the percentage of overlapping mutants showing transcriptional impairment was also relatively low (of the 64 and 65 mutants with p*ALD3*-qV transcriptional defects upon heat shock and osmostress, respectively, 21 were commonly affected by both stress conditions) (Figure 3A; right Venn diagram). These data suggest that no general pattern was identified, thereby supporting the notion that the histone residues required for transcriptional regulation are promoter-specific but also, to a lesser extent, stress-specific. Thus, there is a customization, rather than a universal pattern, of histone residue requirements governed for the specific stress-responsive promoter or depending on the stress that cells are exposed to.

To characterize the nature of the residues whose mutations impaired the transcriptional stress-dependent response, we sorted them by amino acid type, normalizing by the fraction of each amino acid present in the histones (Figure 3B). Of the 20 amino acids, mutations on lysine (K) showed a higher percentage of transcriptional defects in response to stress. It is worth mentioning that serine (S), arginine (R) and glutamine (Q) were also significantly overrepresented in the analysis, although only in a particular stress condition and reporter. This observation suggests that residues that can be post-translationally modified are more susceptible to stress-induced defects in transcription.

Amino acid residues of histones can be found at various sites: buried (inside the histone structure), disk (protein surface that does not contact DNA), lateral (protein surface that contacts DNA), and tail (protruding unstructured region). We analyzed the spatial distribution of mutations leading to defects in response to osmotic and heat stress by sorting the histone residues by position according to the nucleosome crystal structure (32,36). The spatial distribution pattern of the affected residues was similar under osmotic and heat stress, with residues located on the histone surface being the most frequent. Specifically, buried amino acids accounted for only 15–25%, whereas the other 80% were distributed among histone tails, disk and lateral regions (Figure 3C).

To visualize the location of histone residues involved in the transcriptional regulation, we visualized all data obtained from our screenings within the three-dimensional structure of yeast nucleosome, using Pymol Molecular Graphic System and the coordinates from the crystal structure downloaded from the Protein Data Bank (accession number 1ID3) (36) (supplementary 3D molecular graphic files). This approach allowed us to identify clusters of residues that could potentially be transcriptional regulatory regions or serve as structural platforms for the recruitment of histone-modifying enzymes. One example of such regions was the nucleosome acidic patch H2A-E57, H2A-E65 and H2B-E116, where the negative charge is required to define chromatin properties and composition (48). We also identified the H2A-R43, H2A-R78, H2B-R36, H3-R83 and H3-R63 arginines that contact DNA and whose mutations were described previously to interfere with transcription (49). Another known group of residues derived from our screening were the amino acids from G14 to K20 in the N-terminal tail of histone H4, which are necessary for proper H3-K79 methylation (50,51).

### The phosphomimetic mutation of H4-S47 is required for proper activation of genes responsive to osmostress

After global analysis of the histone residues required for proper transcriptional response to stress, we next focused on specific examples. To narrow down the candidates, we assessed their growth phenotype upon heat or osmotic stress and identified those who had greater defects in cell growth. We then discarded the histone residues that were buried within the nucleosome structure and the ones that cannot be post-translationally modified due to their biochemical properties. Of these, we chose to validate the H4 serine 47 (H4-S47) and H4 threonine 30 (H4-T30), as a proof of concept of the potential of the genetic screenings performed. To confirm the existence of these two phosphorylation sites, we performed in-depth phosphoproteomics experiments on WT yeast cells. Out of three biological replicates, we confidently identified 20 530 unique unambiguously localized phosphorylation sites (MaxQuant localization probabilities ≥ 0.75) on 42 635 unique phosphopeptides (Supplementary Table S2). More importantly, both T30 and S47 of the H4 protein were found to be phosphorylated, thereby confirming the *in vivo* phosphorylation of these sites (Supplementary Figure S1).

**Figure 4.**
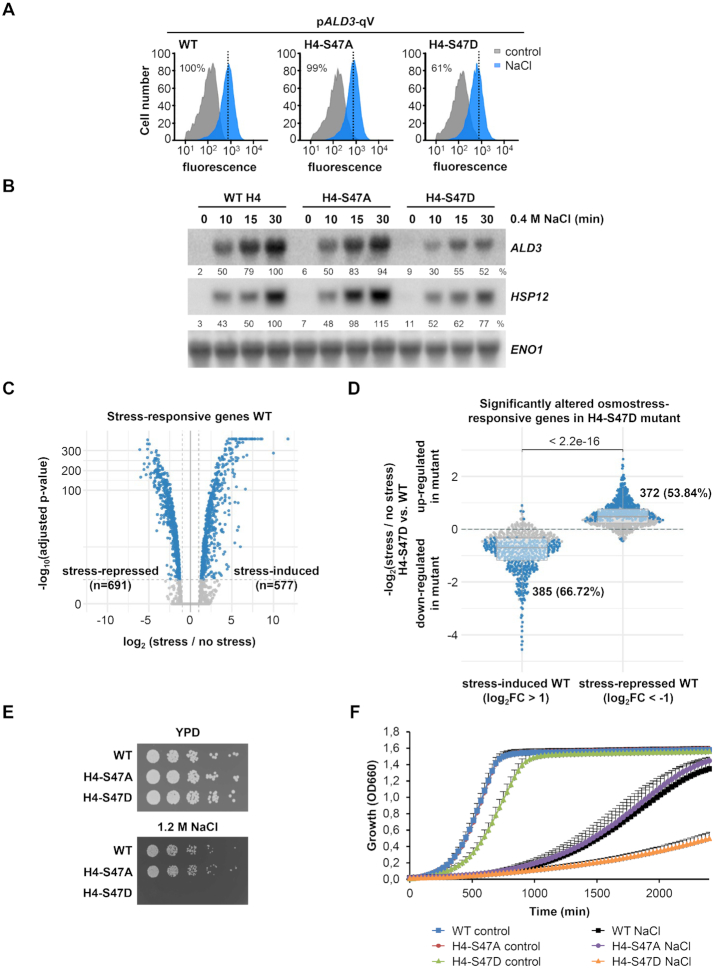
H4-S47D phosphomimetic mutant exhibits transcriptional and growth defects upon osmostress. (**A**) Fluorescence of the quadruple-Venus (qV) reporter driven by *ALD3* promoter was measured by flow cytometry before (control; gray) and after osmostress (0.4 M NaCl for 45 min, blue) in wild-type (WT) and the mutant H4-S47A and H4-S47D strains. Dotted black lines indicate the level of transcription of the WT in response to stress. Fluorescence intensity under stress was quantified with respect to the WT strain and the percentages are indicated. (**B**) WT and the indicated histone mutant strains were subjected to osmostress (0.4 M NaCl) for the indicated length of time. Total mRNA was assayed by northern blot with radiolabeled probes for *ALD3* and *HSP12* (stress-responsive genes) and *ENO1* (as loading control). RNA quantification is expressed in percentage as the ratio of mRNA levels normalized by *ENO1* and taking the value of maximum gene expression of the WT strain as 100% reference. (**C**) Volcano plot showing significantly altered gene expression in WT strain upon stress (0.4 M NaCl, 15 min). Dashed vertical lines mark the imposed log_2_ fold change threshold of 1 (up-/down-regulation), whereas the dashed horizontal line indicates an adjusted *P*-value of 0.05. (**D**) Differences in the transcriptional stress-activated and stress-repressed response defined in (**C**) between H4-S47D mutant and WT strains. Horizontal lines in the boxplots indicate the median, dashed gray lines indicate a relative absolute fold change of 1. The indicated *P*-value is based on a Wilcoxon-test, while the highlighted numbers represent genes significantly altered by the mutant in response to osmostress compared to non-stress conditions (blue dots). (**E**, **F**) Cell growth analysis in response to osmostress. WT and the indicated histone mutant strains were grown to mid-log exponential phase in YPD, serially diluted (2-fold), spotted onto YPD plates with and without 1.2 M NaCl and grown at 30°C for 3–5 days (E). Exponential phase cell cultures were diluted in YPD or YPD 1.2 M NaCl and grown at 30°C with constant agitation for 40 h. Cell density (OD_660_) was measured using a Synergy H1 Hybrid Reader (BioTek). Growth curve data are the means ± SD of a minimum of three technical replicates from a representative experiment (F).

For H4-S47, the mutation of the serine to aspartic acid (H4-S47D), which confers a negative charge mimicking a phosphorylation, resulted in a reduced expression of p*ALD3-qV* upon osmostress, whereas the non-phosphorylatable alanine substitution (H4-S47A) had no effect on the expression of this reporter (Figure 4A). To validate the results for p*ALD3-qV*, we used northern blots to assess the mRNA levels of endogenous osmostress-dependent genes (*ALD3* and *HSP12*) in wild-type, H4-S47A and H4-S47D mutant strains. As expected, upon stress, the phosphomimetic mutant showed a reduced expression of *ALD3* and *HSP12* compared to wild-type cells, while gene expression of the non-phosphorylatable mutant was similar to that observed in wild-type cells (Figure 4B and Supplementary Figure S2A).

To further depict the effect of mimicking the H4-S47 phosphorylation state on osmostress-induced gene transcription, we characterized the genome-wide gene expression in non-stressed and stressed conditions by performing RNA-seq in wild-type and H4-S47D mutant cells. Upon osmostress (0.4M NaCl, 15 min), the expression profiles of 1268 genes were found to be altered at least 2-fold (FDR < 0.05) in the wild-type strain (577 stress-induced and 691 stress-repressed) (Figure 4C and Supplementary Figure S3B). Of note, almost 70% of the genes (385 out of 577) that were induced by osmostress in wild-type cells exhibited a significantly smaller induction in H4-S47D mutant cells (Figure 4D). In line with the previous results, *ALD3* and *HSP12* were in this group of 385 genes. To further characterize these genes, we performed functional enrichment analyses based on Gene Ontology, KEGG and REACTOME using the gProfileR package (39). However, no significant term enrichment was found when comparing mutant-affected genes against all stress-induced affected genes. These results led us to conclude that the phosphomimetic H4-S47D mutant is required for proper activation of genes responsive to osmostress. Additionally, a notable percentage of stress-repressed genes (54%) showed a significant de-repressed response in H4-S47D cells (Figure 4D). In contrast to the stress-induced genes, we found significantly enriched terms related to ribosome biogenesis and rRNA processing (Supplementary Table S3A). It is worth mentioning that, under non-stressed conditions, gene expression was up-regulated in these cells, with only a few genes significantly repressed versus the wild-type strain (FDR < 0.05 and at least 2-fold difference) (Supplementary Figure S3A). To further elucidate that gene expression is both stress- and promoter-specific (as previously shown for the *ALD3*, *HSP82* and *STL1* promoters), we selected target genes of the Msn2/4 and Hsf1 transcription factors and used RNA-seq data to quantify their transcriptional profiles across the different conditions. Although these genes are targeted by the same transcription factors, they show diverse transcriptional responses within the same condition, as well as across different stresses. These observations imply that promoter-specific regulation is a key mechanism through which gene expression is fine-tuned under stress (Supplementary Figure S4). Thus, histone mutations triggered context-dependent activating or repressing roles in transcription.

Transcription plays a crucial role in long-term stress adaptation and protection against future stresses (reviewed in (1)). Thus, to assess the relevance of the H4-S47D mutant and its transcriptional alteration on cell growth upon osmostress, we monitored cellular growth in the presence of high osmolarity (1.2 M NaCl). The non-phosphorylatable H4-S47A mutant did not show major growth differences compared to wild-type cells in response to osmostress; in contrast, mimicking the phosphorylation of this histone site (H4-S47D mutant cells) clearly impaired growth under conditions of high osmolarity both in plates and liquid cultures (Figure 4E and F). Of note, the phosphomimetic mutant already showed a slight decrease in cell growth under basal conditions.

### Cla4 and Ste20 PAKs phosphorylate H4-S47 and regulate the transcription of osmo-responsive genes upon stress

**Figure 5.**
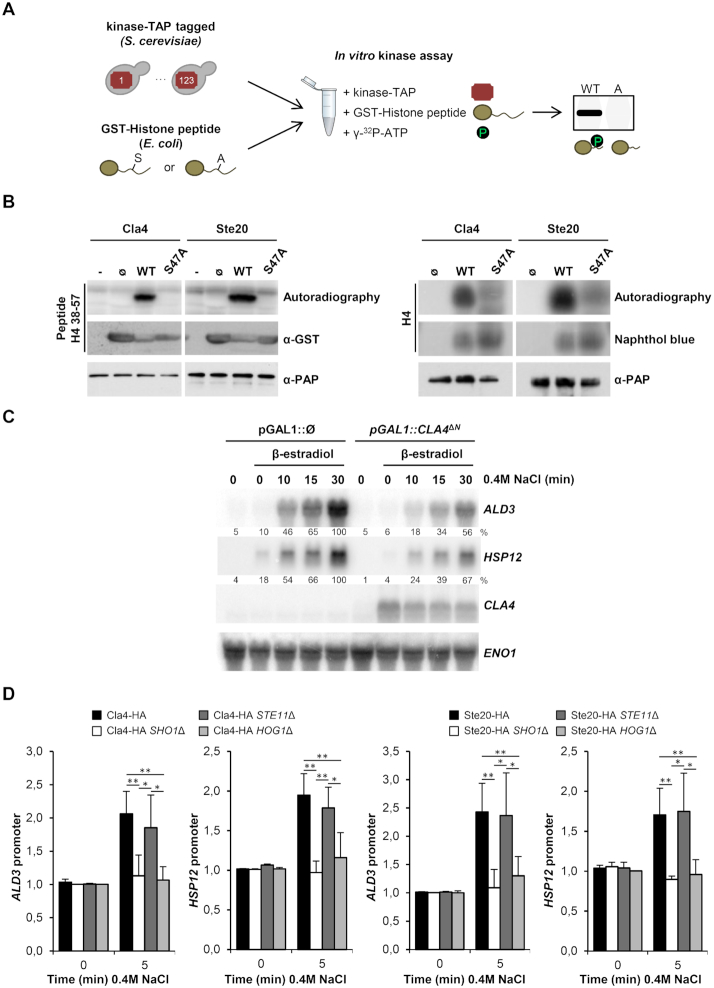
Cla4 and Ste20 PAKs phosphorylate H4-S47 and regulate osmo-responsive transcription. (**A**) Experimental strategy to assess the phosphorylation of histone residue H4-S47 *in vitro*. 123 TAP-tagged kinases were purified from yeast cells and individually assayed with wild-type (WT) and a mutated version (A) of GST-H4 histone peptides purified from *E. coli*. (**B**) Cla4 and Ste20 phosphorylate H4-S47 *in vitro*. Left panel: Fused GST-short peptides (amino acids 38–57) containing the H4-S47 (WT) or the mutated version (S47A) were used as substrates. Empty GST protein (Ø) and no substrate (–) were used as negative controls. Right panel: Purified full-length WT histone H4 or the S47A mutant were used as substrates. Radiolabelled peptides or full-length histones were resolved by SDS-PAGE, transferred to a nylon membrane, and detected by autoradiography. TAP-tagged kinases and GST-tagged histone peptides were detected by western blot. Full-length histones were detected by naphthol Blue. (**C**) Constitutive activation of Cla4 mimics the osmo-responsive transcriptional defect of H4-S47D mutant cells. mRNA levels of stress-responsive genes (*ALD3* and *HSP12*) upon osmostress (0.4 M NaCl) for the indicated length of time were assessed by northern blot in cells harboring a constitutively activated version of Cla4 (p*GAL1::CLA4^Δ^^N^*) or not (p*GAL1:: Ø*), which are inducible by β-estradiol addition. RNA levels were quantified as described in Figure 4B. (**D**) PAKs are recruited to stress-responsive promoters upon osmostress. HA-tagged Cla4 and Ste20 cells were treated with 0.4 M NaCl for the indicated length of time. ChIP against HA was performed to analyze binding to *ALD3* and *HSP12* promoters. Real-time PCR results are shown as the fold induction relative to the non-treated wild-type strain normalized to a telomere internal control. Data are reported as mean ± SD. *P < 0.05, **P < 0.01.

To identify the kinase(s) that could potentially modify the H4-S47 histone residue, we performed an unbiased kinome screening in which we tested 123 tagged-purified yeast protein kinases in an *in vitro* kinase assay, using as a substrate a short H4 histone recombinant GST-peptide (amino acids 38–57) carrying either the wild-type or the mutated (S47A) versions (Figure 5A; see Materials and Methods). This screening yielded five kinases which phosphorylated the wild-type H4-S47 peptide (Supplementary Figure S5A). Only three of these (Cla4, Ste20 and Skm1) did not phosphorylate the H4-S47A mutant peptide. The three kinases belong to the same family known as PAK kinases. Since Cla4 and Skm1 are paralogs in yeast, we further characterized Cla4 and Ste20, both homologs of the mammalian PAK (p21-activated kinase) family (Figure 5B, left panel). Additionally, both kinases phosphorylated purified full-length histone H4 *in vitro* but not the full-length H4-S47A mutant (Figure 5B, right panel), thereby indicating that H4-S47 is a *bona fide* target of Cla4 and Ste20. Of note, in mammals, PAK2 kinases were shown to target H4-S47 residue (52,53), which shows a conservation of this particular histone modification from yeast to mammals (see discussion). In yeast, it has been reported that kinases Cla4 and Ste20 target several genes under basal conditions (40). However, we did not find a significant enrichment for these kinase target genes among the genes identified in our RNA-seq analysis of the H4-S47D mutant (Supplementary Table S4). Of note, Cla4 and Ste20 target genes were defined in non-stress conditions, so it is possible that different targets not considered in our analysis may arise upon stress.

The phosphomimetic H4-S47D mutant displays reduced activation of osmostress-dependent genes. We therefore tested whether the activation of PAKs also triggers a similar transcriptional phenotype upon osmostress. To this end, we expressed an active version of Cla4 that contains a deletion of its N-terminal region (*CLA4*^Δ^*^N^*) (54) under the control of the *GAL1* promoter, using the pADGEV plasmid (to induce the expression of *GAL1* in the presence of estradiol) (37). In the presence of estradiol, N-terminal deleted Cla4 was expressed and mRNA levels of the stress-responsive genes *ALD3* and *HSP12* were reduced compared to control cells (Figure 5C and Supplementary Figure S2B) and similar to those in the H4-S47D mutant strain (Figure 4B). It is worth mentioning that inducing Cla4^ΔN^ overexpression had no transcriptional effect on the non-phosphorylatable H4-S47A mutant (Supplementary Figure S6). This result suggested that the PAKs regulate the activation of stress-responsive genes by phosphorylating H4-S47.

Using chromatin immunoprecipitation (ChIP) assays, we next analyzed the binding of the HA-tagged PAKs to osmostress-dependent promoters. Cla4 and Ste20 were recruited to stress-specific loci (*ALD3* and *HSP12* promoters) in response to osmostress (Figure 5D). In response to osmostress, the high osmolarity glycerol (Hog1) MAPK, homolog of mammalian p38, is activated by two upstream independent signaling mechanisms: the transmembrane histidine kinase Sln1 and the Sho1 osmosensing branch (reviewed in (2)). The two osmosensing branches of the HOG pathway converge at Hog1, which is essential for gene expression in response to osmostress (reviewed in (55)). Of note, in *HOG1*Δ cells, Ste20 and Cla4 were not bound to *ALD3* or *HSP12* promoters (Figure 5D). Upon stress, Sho1 activates the Ste11 MAP3K through the integral transmembrane protein Opy2, the GTPase Cdc42 and the PAKs Ste20 and Cla4 (2). To identify the signaling requirements for chromatin binding to PAKs, we monitored chromatin recruitment in *SHO1*Δ and *STE11*Δ mutant cells. In *SHO1*Δ cells, both Cla4 and Ste20 lost their binding to the osmostress-dependent promoters, whereas recruitment of the PAKs in *STE11*Δ cells was similar to that observed in wild-type cells (Figure 5D). Overall, these data suggest that once activated upon stress, the PAK kinases associate to stress-responsive genes. To further assess the effect of these kinases on transcriptional regulation, we artificially tethered Cla4^ΔN^ kinase to the non-stress responsive *ADH1* promoter introducing 8 LexA operators and expressing a plasmid harboring the fusion protein HA-tagged LexA DNA binding domain with the Cla4^ΔN^ kinase under the inducible expression system ADGEV. Upon induction of the pLexA-HA-Cla4^ΔN^ with estradiol, there was a down-regulation of *ADH1* mRNA when compared to the control (HA-tagged LexA strain) (Supplementary Figure S7A). To demonstrate the kinase tethering, we analyzed the binding of this fusion protein to the *ADH1* promoter by means of ChIP (Supplementary Figure S7C). These results further highlight the down-regulatory effects of the PAK2 kinases on local gene expression.

### Ste11 phosphorylates H4-T30 to regulate transcription upon stress

**Figure 6.**
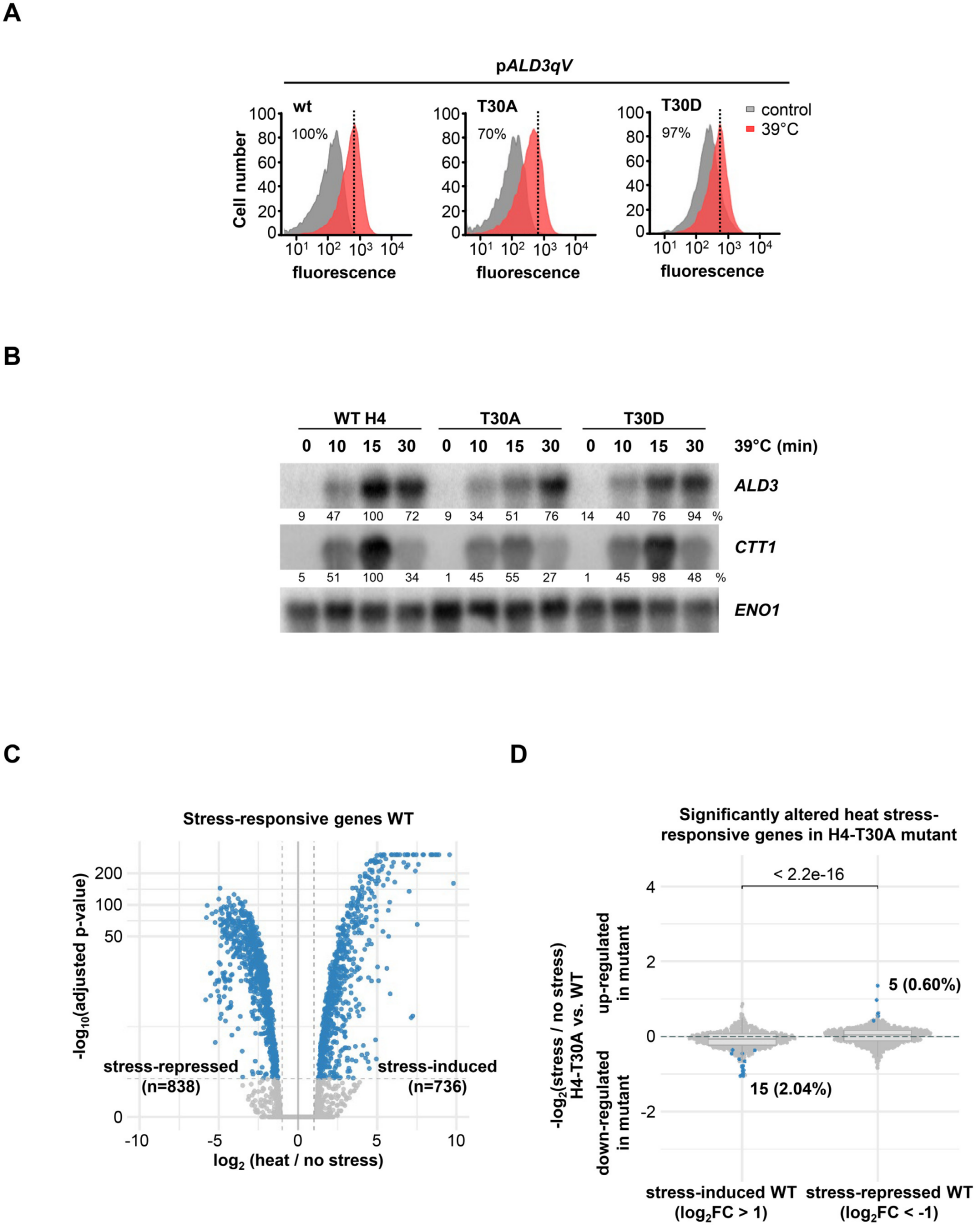
H4-T30 is required for proper transcriptional response to heat stress. (**A**) Fluorescence of the quadruple-Venus (qV) reporter driven by *ALD3* promoter was measured by flow cytometry before and after heat stress (39°C for 45 min) in wild-type (WT), H4-T30A and H4-T30D mutant strains. Dotted black lines represent the level of transcription of the WT upon stress. Fluorescence intensity in stressed condition was quantified with respect to the WT strain, as indicated in percentages. (**B**) Histone mutant strains were subjected to heat stress (39°C) for the indicated length of time. Total mRNA was assayed by northern blot with radiolabeled probes for *ALD3* and *CTT1* (stress-responsive genes) and *ENO1* (as loading control). RNA levels were quantified as described in Figure 4B. (**C**) Volcano plot showing significantly altered gene expression in WT strain upon stress (39°C, 10 min). Dashed vertical lines mark the imposed log_2_ fold change threshold of 1 (up-/down-regulation), whereas the dashed horizontal line indicates an adjusted *P*-value of 0.05. (**D**) Differences in the stress-activated and stress-repressed transcriptional responses defined in (C) between H4-T30A and WT strains. Horizontal lines in the boxplots indicate the median, and dashed gray lines indicate a relative absolute fold change of 1. The indicated *P*-value is based on a Wilcoxon-test, while the highlighted numbers represent genes significantly altered by the mutant in response to heat stress compared to non-stress conditions (blue dots).

Another residue of interest from the transcriptional screenings was the H4 threonine 30 (H4-T30), an as of yet novel target residue. The mutation of this residue to the non-phosphorylatable alanine (H4-T30A) resulted in reduced expression of *pALD3-qV* upon heat stress, whereas the phosphomimetic mutant (H4-T30D) had no effect on the expression of this reporter (Figure 6A). Correspondingly, the expression of endogenous stress-dependent genes *ALD3* and *CTT1* in H4-T30A cells was diminished upon heat stress (Figure 6B, Supplementary Figures S2C and S8). Of note, H4-T30 did not contribute to transcription in response to other stresses involving Msn2/4 transcription factors, such as osmotic stress or nutrient starvation (Supplementary Figure S9). In non-stressed conditions, the wild-type and H4-T30A mutant strains showed a very similar expression pattern, as measured by RNA-seq analysis (Supplementary Figure S3C, left panel). Under heat stress (39°C, 10 min), a large fraction of genes showed altered gene expression (838 and 736 genes down- and up-regulated, respectively; FDR < 0.05 and at least 2-fold difference). However, only a small group of these heat stress-responsive genes was significantly altered in the H4-T30A mutant when compared with wild-type cells: 15 genes showing less induction upon stress, among them *ALD3*, while 5 genes showed a de-repressed expression (Figure 6D and Supplementary Figure S3D).

**Figure 7.**
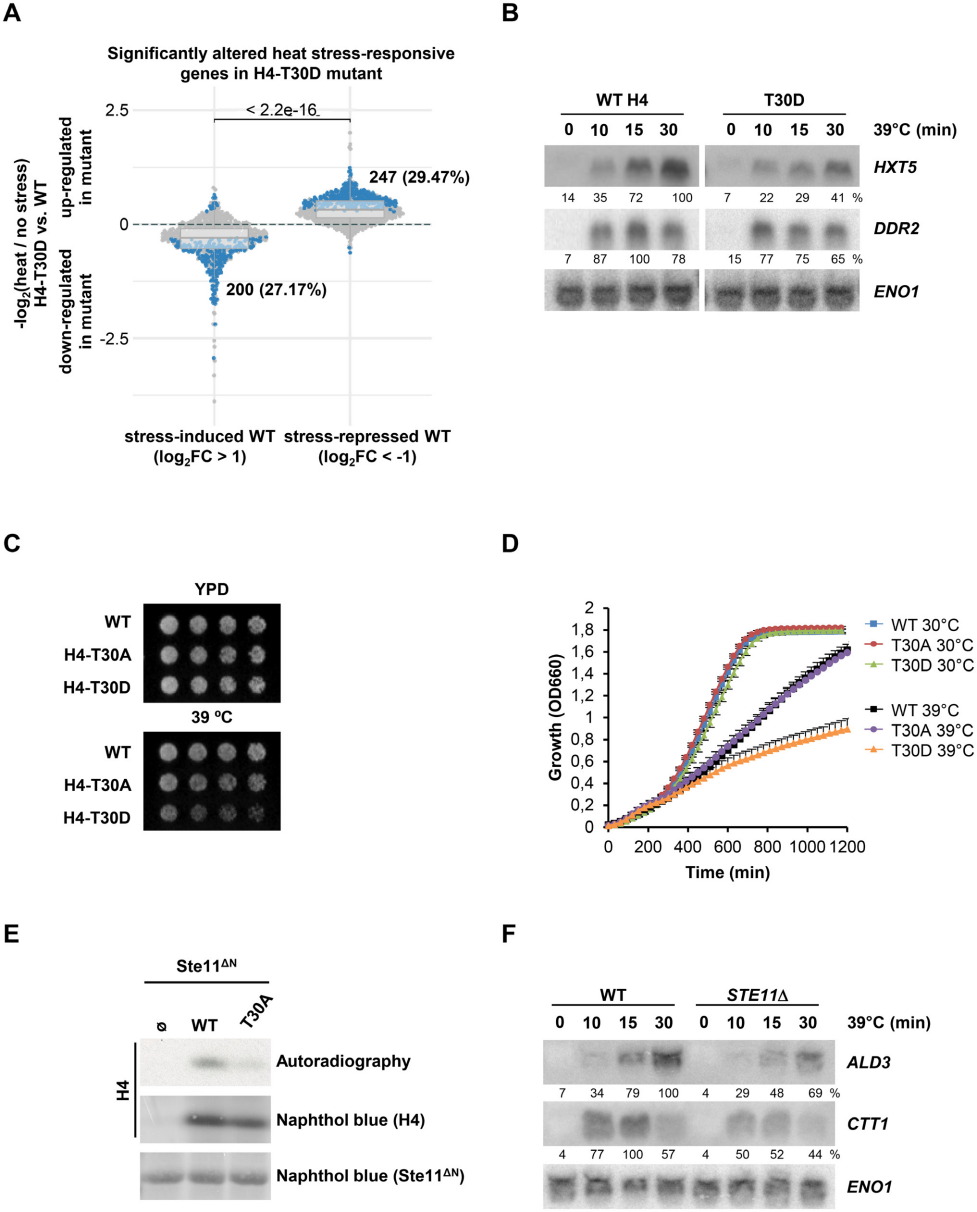
H4-T30D phosphomimetic mutant triggers transcriptional and growth defects in response to heat stress. (**A**) Differences in the stress-activated and stress-repressed transcriptional responses defined in (C) between H4-T30D and wild-type (WT) strains. Horizontal lines in the boxplots indicate the median, and dashed gray lines indicate a relative absolute fold change of 1. The indicated *P*-value is based on a Wilcoxon-test, while the highlighted numbers represent genes significantly altered by the mutant in response to heat stress compared to non-stress conditions (blue dots). (**B**) Histone mutant strains were subjected to heat stress (39°C) for the indicated length of time. Total mRNA was assayed by northern blot with radiolabeled probes for *HXT5* and *DDR2* (stress-responsive genes) and *ENO1* (as loading control). RNA levels were quantified as described in Figure 4B. (**C**, **D**) Cell growth analysis upon heat stress. For cell dots, WT and the indicated histone mutant strains were grown to mid-log exponential phase in YPD, serially diluted (2-fold), spotted onto YPD plates, and then grown at 30°C or 39°C for 3–5 days (C). For growth curves, exponential phase cell cultures were diluted in YPD and grown at 30°C or 39°C with constant agitation for 20 h. Cell density (OD_660_) was measured using a Synergy H1 Hybrid Reader (BioTek). Data are the means ± SD of a minimum three technical replicates from a representative experiment (D). (**E**) Ste11 phosphorylates the H4-T30 residue *in vitro*. *In vitro* kinase assay using a constitutively activated version of Ste11 (Ste11^ΔN^) and full-length WT or H4-T30A histone versions as substrates. Radiolabeled peptides were resolved by SDS/PAGE, transferred to a nylon membrane and detected by autoradiography. Both GST-tagged Ste11^ΔN^ and full-length histone were detected by Naphthol blue. (**F**) *STE11*Δ mutant cells mimic the transcriptional defect of the non-phosphorylatable H4-T30A mutant upon heat stress. mRNA levels of stress-responsive genes upon heat stress (39°C) were assessed by northern blot in WT and *STE11*Δ strains. RNA levels were quantified as described in Figure 4B.

To assess whether the modification of this histone residue triggered a more severe transcriptional defect, we performed RNA-seq assays again but comparing the phosphomimetic T30D mutant with wild-type cells. In non-stressed conditions, both strains had a similar expression pattern, although a minor set of genes was de-repressed (Supplementary Figure S3C, right panel). Upon heat stress, around 25–30% of genes were significantly down- or up-regulated in the H4-T30D mutant *versus* wild-type cells (Figure 7A and Supplementary Figure S3D). Up-regulated genes were enriched for terms related to cytoplasmic translation, rRNA export, ribosome and non-sense mediated decay (Supplementary Table S3B), while down-regulated genes showed enrichment for terms related to amino acid biosynthesis (Supplementary Table S3C). Northern blot analysis validated some of the altered genes, such as *HXT5* or *DDR2*, whose expression is dependent on the phosphomimetic mutant on H4 in response to heat stress (Figure 7B and Supplementary Figure S2D). Thus, these genome-wide analyses allowed us to unmask a severe phenotype of the T30D, while demonstrating the limited effect of the T30A mutation. Our findings suggest that the T30 mutation on H4 is critical in the regulation of the transcription of heat stress-responsive genes upon stress. Moreover, as shown before (Supplementary Figure S4), the expression of individual genes is controlled by an assortment of transcription factors and specific histone modifications that fine-tune the promoter- and stress-specific regulation of gene expression under stress. To characterize the effects of mimicking or avoiding the phosphorylation of H4-T30, we performed cell growth assays under heat stress. In agreement with the severity of its transcription defects, H4-T30D impaired growth at 39°C compared to wild-type cells (Figure 7C and D).

To identify the kinase(s) that potentially regulate T30 phosphorylation on H4, we performed a similar biochemical approach as before (Figure 5A) and assayed *in vitro* the entire kinome with H4 histone recombinant GST-peptides (amino acids 2–46 WT or T30A) as substrates. This kinase screening yielded 17 kinases that phosphorylated the wild-type H4-T30 (Supplementary Figure S5B). Of these kinases, only 2 (Ste11 and Cdc5) did not phosphorylate the alanine mutant H4-T30A peptide. We opted to further characterize the Ste11 MAP3K as a putative kinase to phosphorylate H4-T30. Indeed, purified yeast active Ste11 (Ste11^ΔN^ (42)) phosphorylated *in vitro* full-length histone H4 but not the H4-T30A mutant (Figure 7E). We then checked Ste11 kinase target gene enrichment among the histone H4-T30D mutant affected genes defined by our RNA-seq data but no significant enrichment was found based on the public Ste11 target gene list (40) (Supplementary Table S4). The Ste11 MAP3K has been involved in the heat stress response (56–59). Next, we tested whether the deletion of *STE11* down-regulates gene expression similar to the non-phosphorylatable mutant of T30 on H4 upon heat stress. Since we found that expression of *ALD3* and *CTT1* was reduced in the T30A mutant, we assessed whether mutation of Ste11 triggered a similar transcriptional impairment. As shown by northern blot, mRNA levels of the stress-responsive genes *ALD3* and *CTT1* were decreased in the *STE11Δ* strain compared to control cells (Figure 7F and Supplementary Figure S2E), correspondingly to H4-T30A mutant strain (Figure 6B), thereby suggesting that Ste11 regulates the activation of heat-responsive genes by targeting H4-T30. To further assess the effect of the Ste11 kinase in non-heat-responsive genes, we artificially tethered Ste11^ΔN^ kinase to the non-stress responsive *ADH1* promoter as before. Upon induction of the pLexA-HA-Ste11^ΔN^ with estradiol, there was a down-regulation of *ADH1* mRNA when compared to the control (HA-tagged LexA strain) (Supplementary Figure S7B). To demonstrate the kinase tethering, we analyzed the binding of this fusion protein to the *ADH1* promoter by means of ChIP (Supplementary Figure S7C). Taken together, we found a new modifiable histone residue relevant for the transcriptional response upon stress.

## DISCUSSION

The transcriptional landscape of cells changes dramatically in response to extracellular stimuli. Histone PTMs play a crucial role in regulating gene expression, thereby facilitating such transcriptional reprogramming (20,32,60,61). In this study, we globally assessed the histone requirements for a proper transcriptional response to osmostress and heat shock. Using genome-wide genetic screenings, we identified more than 200 histone point mutations that are required for the correct activation of one or more stress-responsive gene reporters. Of note, some of the histone mutants uncovered corresponded to histone residues whose PTMs have already been described to regulate transcription (e.g., H3-K4me3 (62,63), H3-K36me3 (64), H4-K20me (65), H3-K56ac (28), H3-K122ac (23) and H4-K31ub (66)). These examples validate the capacity of our genetic approach to reveal novel histone residues relevant for transcriptional regulation.

In general terms, the histone residues whose mutations triggered inappropriate transcriptional activation of the stress-dependent reporters were distributed relatively equally among the four histones. However, on histones H2A and H2B, mutants with higher basal expression on the p*HSP82-qV* reporter were overrepresented. In contrast to *ALD3* and *STL1*, where specific transcription factors are recruited only in response to stress, the transcription factor Hsf1 binds to the *HSP82* promoter in non-stress conditions (67,68). This binding confers an open conformation to the promoter, thereby allowing the transcriptional machinery to fire within seconds of a shift in temperature (69). It is also known that H2A/H2B dimers are more easily displaced from the nucleosome (70,71). Thus, point mutations in H2A/H2B in some specific promoters might cause changes in the nucleosome structure and relax the nucleosome blockage, thereby increasing the basal expression of the fluorescent reporter.

In addition to the list of specific histone mutants with a transcriptional phenotype, this study has revealed some general features regarding the involvement of histones in the massive and rapid reorganization of the transcriptional program in response to stress. First, the requirements of histone residues seem to be gene-specific, since there was a relatively poor overlap between the different reporters (p*ALD3-qV vs*. p*HSP82-qV* upon heat stress and p*ALD3-qV vs*. p*STL1-qV* upon osmostress). This minimum overlap could be explained by the fact that each promoter is regulated by distinct transcription factors, Msn2/4 for *ALD3*, Hsf1 for *HSP82*, and Hot1 and Sko1 for *STL1* (8,9,72–75), and these factors have specific mechanisms through which to initiate transcription. For instance, the Rpd3 deacetylase histone complex is recruited to Msn2/4- but not to Hsf1-dependent genes upon heat stress (10,76), and the interactions of some chromatin remodelers, such as SWI/SNF, RSC and ISWI with these promoters are known to differ (11,12,77,78). Second, the landscape of histone residues depends on the type of stress (heat stress versus osmotic stress in p*ALD3-qV*). This distinction might be due to the fact that each stimulus is governed by specific pathways with different dynamics, thereby resulting in distinct chromatin signatures for optimal transcription. However, 19 out of 21 histone residues from the overlap between stresses were previously reported to render transcriptional defects (26,32,79), thereby suggesting that they belong to common regulatory mechanisms or are involved in fundamental processes that regulate nucleosome stability/dynamics, regardless of the environmental stresses. Some of these histone residues, such as the H3-K4, H4-K91 and H2A-I112, are known to be relevant for histone biology (80–82) while others remain unreported. Third, most of the residues whose mutations triggered an impairment of transcriptional initiation were located in exposed regions of the nucleosomes, either in the histone tails or on the surface of the histones. These results are in agreement with several reported examples describing new modifications in the globular domains of histones (23–25) and provide further insight into how histone modification biology regulates transcriptional processes. In addition to the location of a single residue, the screenings also highlighted the importance of the surrounding context, since there were series of consecutive residues (modifiable or not) with transcriptional defects. These regions could have relevant functions, serving as docking sites for histone modifiers, creating an appropriate context for a catalytic site, or simply structuring the whole histone. Finally, potentially post-translationally modifiable amino acids such as lysine and serine are overrepresented as histone mutants with transcriptional defects. This finding opens a novel framework through which to characterize new histone PTMs required for transcriptional regulation upon the reprogramming of dynamic gene expression. Indeed, some specific histone modifications have larger effects on the dynamics of gene expression than on steady-state transcription (28–31). Nevertheless, it should be taken into account that mutations of amino acids that are either charged or polar might have dramatic effects on protein folding and nucleosome assembly. Moreover, non-modifiable residues were also highly represented in our screenings. These types of hydrophobic amino acids are frequently buried or next to buried residues in folded histones, and they might be more likely to alter nucleosome structure.

With the aim to identify novel histone PTMs required for transcriptional stress-response, here we focused on two specific examples of histone mutations taken from the genetic screenings. Initially, we reported a novel role for H4-S47 on transcriptional regulation upon osmostress. The mutation of this serine to aspartic acid, which mimics its phosphorylation, resulted in a severe alteration of gene expression and a cell growth defect in response to osmostress. Moreover, under non-stress conditions, the S47D mutant cells showed significant de-repressed gene expression. H4-S47 is located on the lateral surface of the histone, in close proximity to the DNA strand. Given the position of this residue, it could be speculated that a permanent negative charge could create more unstable nucleosomes, thus causing this up-regulated gene expression pattern. However, upon stress, the phosphomimetic mutation triggered a massive down-regulation of osmostress-induced genes. These observations thus indicate that the activation of gene expression is targeted by H4-S47 phosphorylation. Indeed, both Thr30 and Ser47 of the histone H4 protein were found to be phosphorylated, supporting our data from the histone mutant screenings. Of note, the phosphorylation of other histone residues in basal conditions, which become functionally relevant in non-steady state conditions, such as cellular stress has been reported (83).

By performing an unbiased biochemical screening, we identified Cla4 and Ste20, homologs of the mammalian PAK2 family, as the kinases that phosphorylated H4-S47. Constitutive activation of Cla4 rendered the down-regulation of stress-responsive genes, similar to the effect of the phosphomimetic H4-S47D mutant in stress conditions. PAK-like kinases have been implicated in the signal transduction in the HOG pathway upon stress through the Sho1 branch that includes the Ste11 MAPK3, the latter phosphorylating Pbs2 MAP2K, which in turn activates the Hog1 MAPK (2). Here we demonstrate that the binding of PAK2 kinases to stress-responsive promoters depends on the upstream signaling component Sho1 but not on the downstream signaling of the HOG pathway. Thus, in this scenario, we propose that, in response to stress, Cla4 and Ste20 play a dual role: (i) they phosphorylate Ste11 to propagate signaling through Pbs2 and Hog1, thereby allowing induction of the transcriptional stress-responsive program (55) and (ii) they phosphorylate H4-S47 located in stress-loci in a MAPK cascade-independent manner. This phosphorylation might lead to a negative loop that collaborates with other mechanisms to attenuate stress-responsive gene expression, which is known to be essential for cell survival (12,18,84). This balance would be disturbed by the S47D mutation or hyper-activation of the PAKs, pulling the system into a more repressive state. Of note, Cla4 and Ste20 have been reported to restrict gene expression. For instance, the PAK-like kinases down-regulate the expression of genes involved in sterol uptake by a MAPK-independent pathway (85). Moreover, the phosphorylation of the H4-T80 by Cla4 upon DNA damage (86) and the H2B-S10 by Ste20 during hydrogen peroxide-induced apoptosis (87) have been reported, thus highlighting the importance of these kinases in histone regulatory pathways. Remarkably, in human cells, PAK2 kinases phosphorylate H4-S47 to enhance the binding of the histone chaperones HIRA, which increase histone repositioning on chromatin (52,53). Whether the phosphorylation of yeast H4-S47 is also required for the recruitment of Hir1, Hir2, Hir3 or HPC2, homologs of the mammalian HIRA complex, has not been addressed to date.

Located in close proximity to the nucleosome entry site, H4-T30 is another potentially modifiable residue whose mutation to a non-phosphorylatable version (H4-T30A) resulted in lower gene expression of the p*ALD3-qV* reporter upon heat stress. Although our RNA-seq data showed that H4-T30A triggered a mild effect on the stress-expression pattern, the phosphomimetic mutant H4-T30D prompted a more severe transcriptional phenotype, as well as a clear impairment of cell growth in response to heat stress. Thus, the phosphorylation balance of H4-T30 seems to be required for proper adaptation to stress. We identified the Ste11 MAP3K as the putative histone kinase of this specific threonine. Indeed, deletion of *STE11* showed transcriptional phenotypes reminiscent of T30A. Ste11 is reported to be an activator of the heat stress response (56–59), although it has never been described as a histone kinase. Of note, the amino acid lying next to H4-T30, H4-K31, is a lysine whose modification regulates transcription (65,88). It remains to be determined whether these two PTMs interact to achieve an optimal transcriptional response.

This study gives a global picture of the specific histone residues required for a massive and rapid transcriptional reprogramming and provides insight into the general features regarding stress-responsive gene regulation by histones. As stated in the introduction, upon stress there is a massive but transient transcriptional outburst. Initially, chromatin remodelers and histone chaperones displace nucleosomes to facilitate the binding of the transcriptional initiation machinery. In this context, histone PTMs and MAPK signaling are relevant to define the nucleosome dynamics and chromatin remodeling in these stress-responsive genes, which ultimately helps to define the timing and intensity of the response (11,14,17). To finely tune the transcriptional response, chromatin remodelers and histone chaperones reposition nucleosomes back while transcription returns to basal levels (12,18). In such dynamic regulation, the phosphorylation of residues H4-S47 and H4-T30 contributes to modulating the transcriptional stress-response. The transcriptional screenings reported here provide valuable information to determine novel histone PTMs, their chromatin modifiers, and their function in stress responses, as we showed with the H4-S47 and T30 residues. Histones are highly conserved among species and, as seen in this study, some modifications and regulatory elements are shared between them. The implications of histone PTMs in the pathogenesis and treatment of human diseases have already been pointed out (56,89,90). Therefore, a deeper understanding of the interface between chromatin regulation and transcriptional control might facilitate better characterization of a wide range of diseases.

## DATA AVAILABILITY

RNA sequencing data have been deposited in the Gene Expression Omnibus (GEO) database (GSE130549).

The mass spectrometry proteomics data have been deposited to the ProteomeXchange Consortium via the PRIDE (46) partner repository with the dataset identifier PXD017134.

## Supplementary Material

gkaa081_Supplemental_Files
