# Supplementary material for: A genetic analysis reveals novel histone residues required for transcriptional reprogramming upon stress
Source: Nucleic Acids Res. 2020 Feb 17;48(7):3455–75. doi: 10.1093/nar/gkaa081 (PMC7144942; doi:10.1093/nar/gkaa081)
Supplement: gkaa081_Supplemental_Files [file gkaa081_supplemental_files.zip › Supplementary Figure 2.0.pdf]

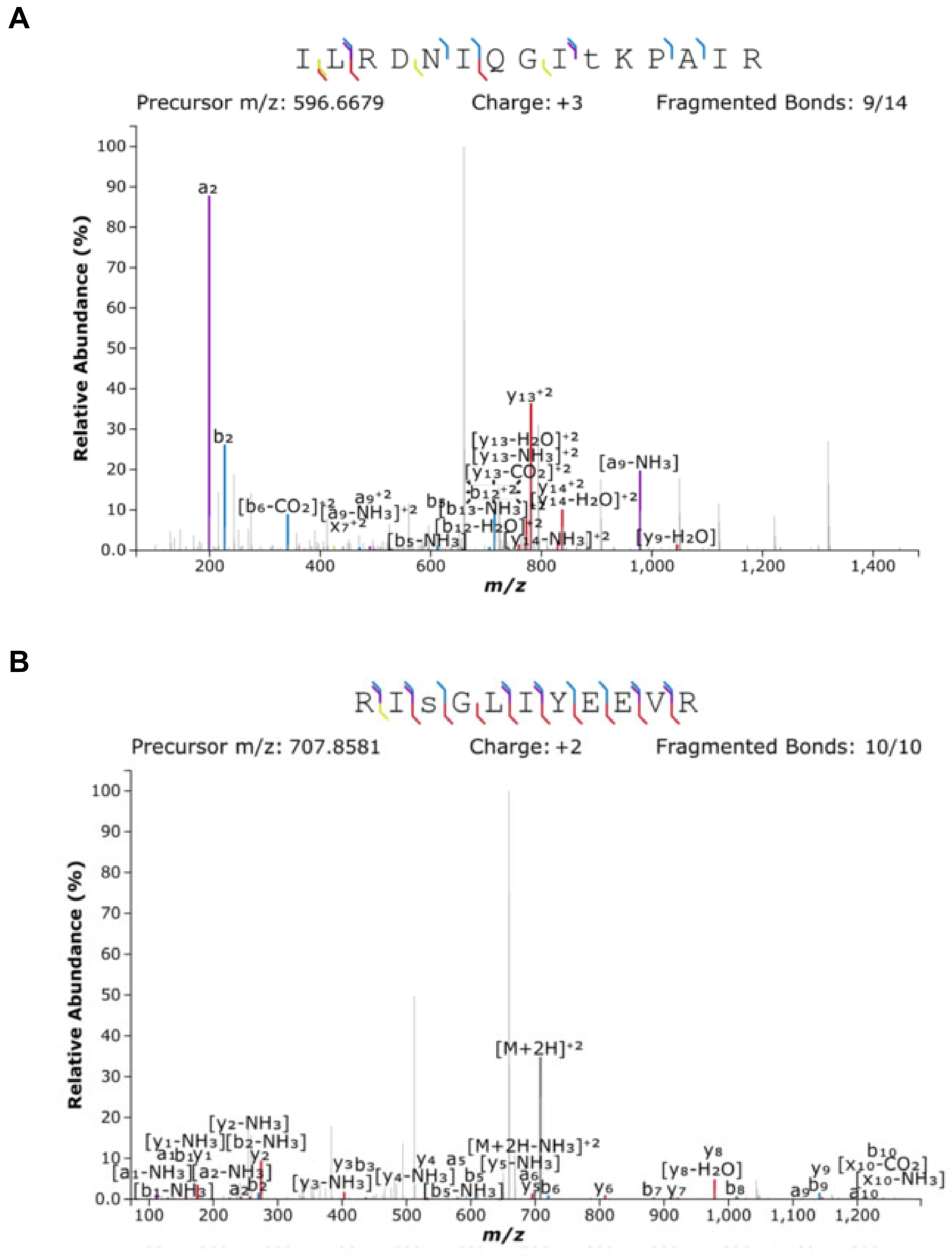

Figure S1: Annotated spectra corresponding to the H4-T30 **(A)** and H4-S47 **(B)** phosphorylation events. Annotation was performed by the Interactive Peptide Spectral Annotator software (IPSA), with a 20 ppm mass tolerance. It has to be noted that commonly observed phosphate neutral losses (HPO_3, H_3PO_4 from precursor or fragments) annotations are not possible with IPSA and are thus not displayed here.

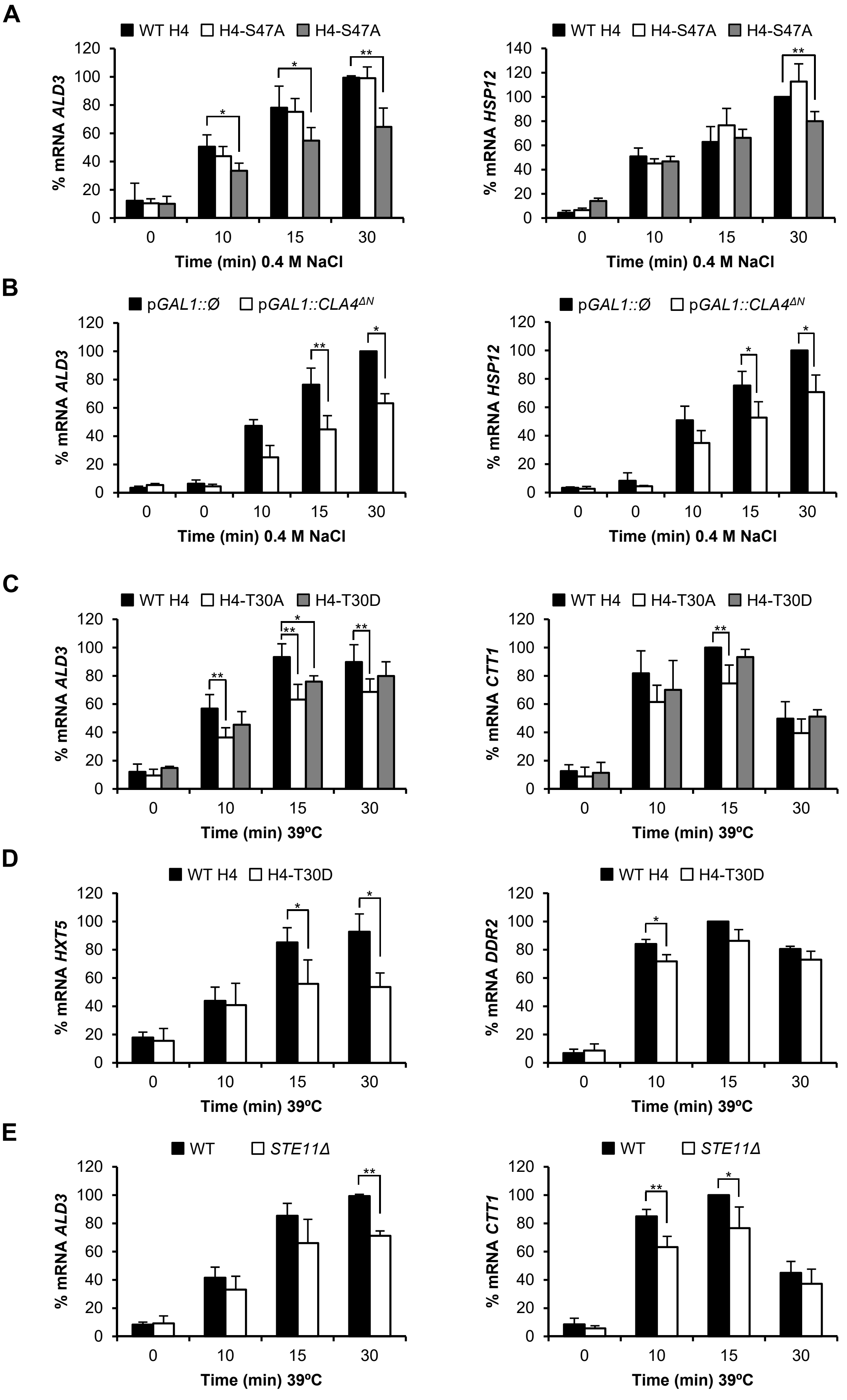

Figure S2. Northern blots quantification. Mean ± SD mRNA expression of at least three experiments as described in Figure 4B **(A)**, Figure 5C **(B)**, Figure 6B **(C)**, Figure 7B **(D)** and Figure 7F **(E)**. $*p < 0.05$, $**p < 0.01$.

A

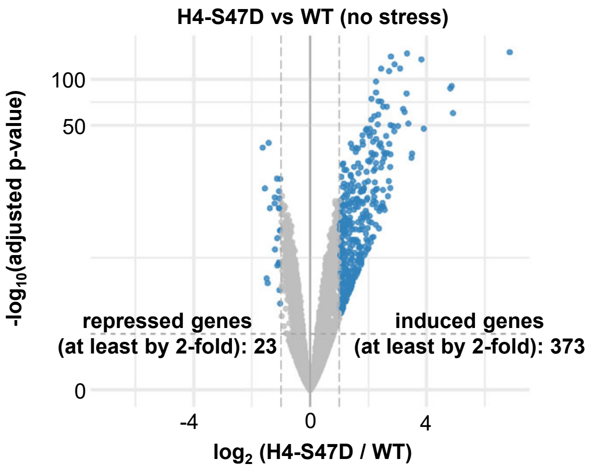

B

	Total genes per group	of which significantly altered in S47D mutant
stress-induced WT (log2FC>1)	577	385 (66.72%)
stress-repressed WT (log2FC<-1)	691	372 (53.84%)
other	5478	852 (15.55%)

C

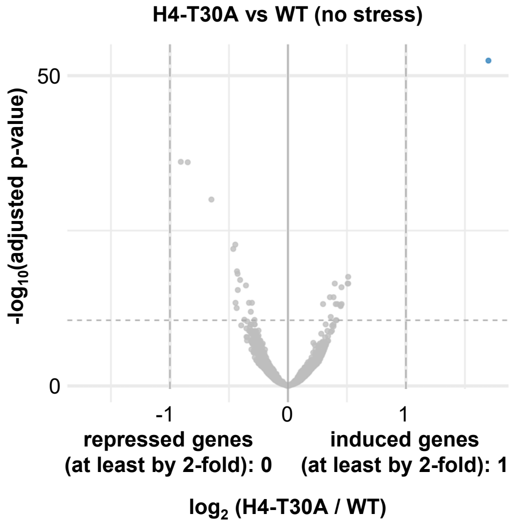

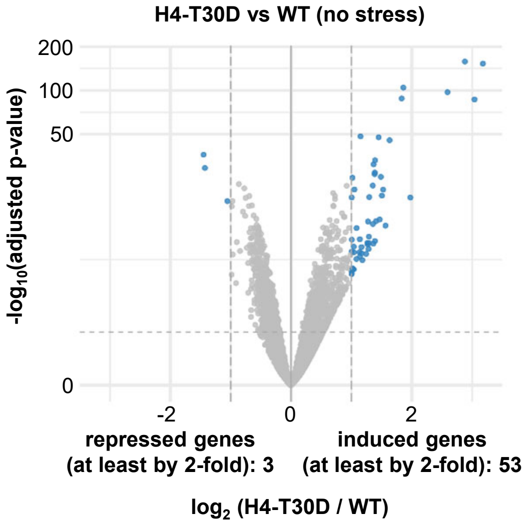

D

	Total genes per group	of which significantly altered in T30A mutant	of which significantly altered in T30D mutant
stress-induced WT (log2FC>1)	736	15 (2.04%)	200 (27.17%)
stress-repressed WT (log2FC<-1)	838	5 (0.60%)	247 (29.47%)
other	5172	14 (0.27%)	292 (5.65%)

Figure S3. RNA sequencing analysis of histone mutant strains upon stress (A) Volcano plot showing significantly altered gene expression of H4-S47D mutant versus wild-type (WT) strains in non-stressed conditions. Dashed vertical lines mark the imposed $\log_2$ fold change threshold of 1 (up-/down-regulation), whereas the dashed horizontal line indicates an adjusted p-value of 0.05. The indicated p-value is based on a Wilcoxon-test. Bold numbers represent genes significantly altered in the H4-S47D mutant compared to WT strains (blue dots). $|\log2FC| > 1$, $FDR < 0.05$. **(B)** Table showing the percentage of stress-responsive genes altered in the H4-S47D mutant. **(C)** Volcano plot showing significantly altered gene expression of H4-T30A (left panel) and T30D (right panel) mutants versus WT strains in non-stressed conditions as in Figure S1A. **(D)** Table showing the percentage of stress-responsive genes altered in H4-T30A and T30D mutants.

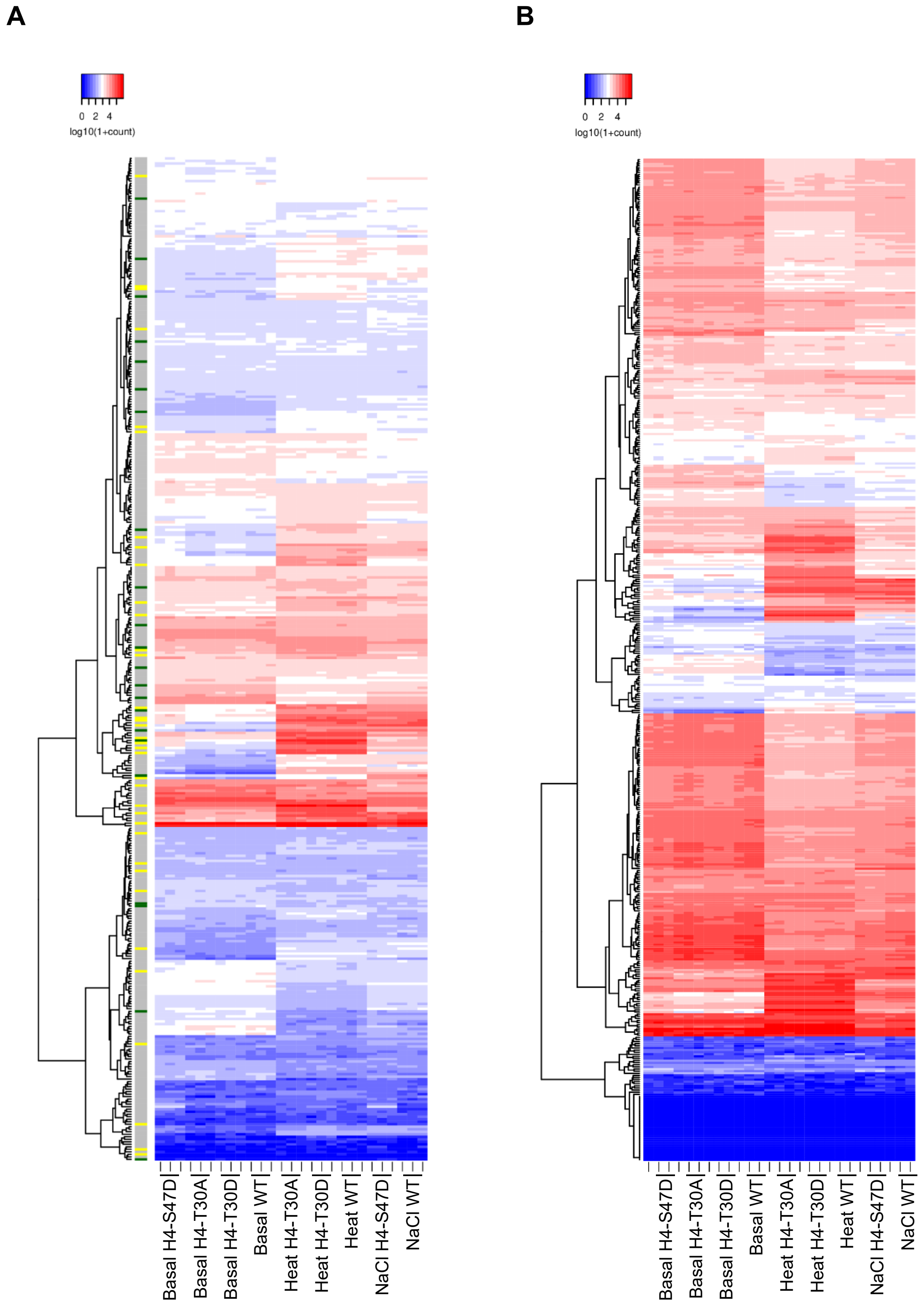

Figure S4. Expression of Msn2/4 and Hsf1-dependent genes on histone mutant strains upon stress. Heatmaps of log10-scaled normalized counts for Msn2/4 **(A)** and Hsf1 **(B)** target genes, with genes displayed in rows and samples in columns. **(A)** Gene color code: in grey, target gene of Msn2; in green, target gene of Msn4; in yellow, target gene of both Msn2 and Msn4. Target gene lists were download from SGD (yeastgenome.org).

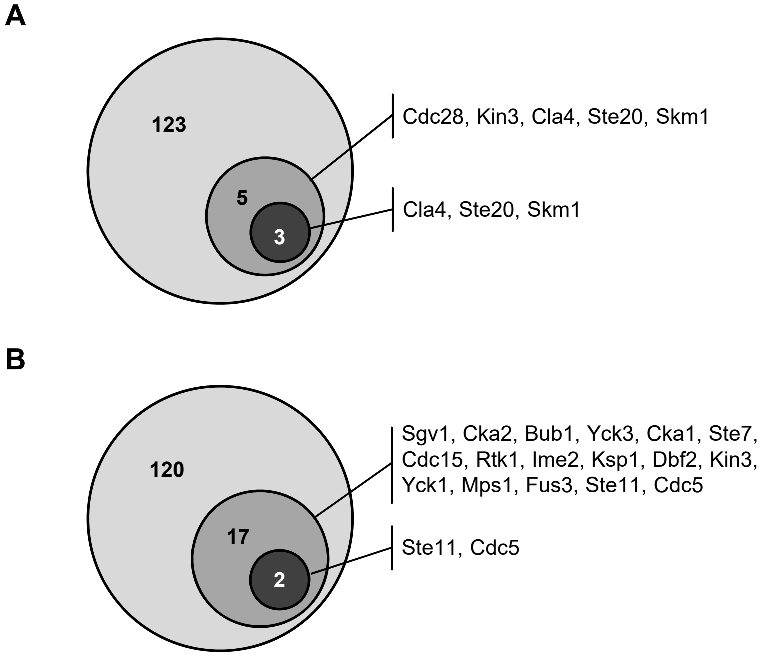

Figure S5. Kinase assay screenings for H4-T30 and H4-S47. (A) The *in vitro* kinase assay screening for H4-S47 identified 5 kinases out of 123 kinases that phosphorylated a fused GST-short histone peptide (amino acids 38 to 57) containing the wild-type H4-S47. From these 5, 3 kinases (Cla4, Ste20 and Skm1) phosphorylated the S47 and not the S47A peptides. **(B)** Similar to (A), an *in-vitro* kinase assay screening identified 17 kinases out of 120 kinases assayed that phosphorylated the wild-type H4-T30 GST-fused short histone peptide (amino acids 2 to 46). 2 (Cdc5 and Ste11) out of these 17 did not phosphorylate the H4-T30A short peptide.

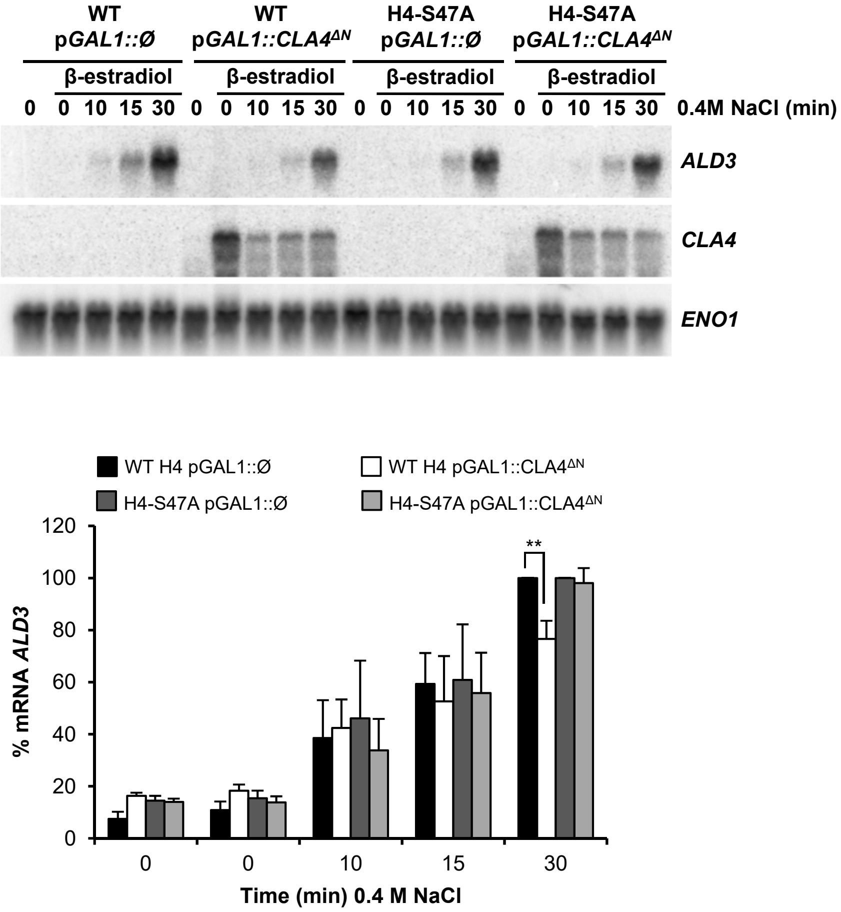

Figure S6. Constitutive activation of Cla4 down-regulates *ALD3* gene expression upon stress in wild-type but not in H4-S47A cells. mRNA levels of stress-responsive gene (*ALD3*) upon osmostress (0.4 M NaCl) for the indicated length of time were assessed by northern blot in H4 WT and H4-S47A mutant strains harbouring a constitutively activated version of Cla4 (p*GAL1::CLA4*$^{\Delta N}$) or not (p*GAL1::Ø*), induced by β-estradiol. RNA quantification is expressed as the ratio of mRNA levels normalized by *ENO1* and taking the value of maximum gene expression of p*GAL1::Ø* strains as 100% reference. The upper panel shows a representative experiment and the lower panel the data of at least three independent experiments reported as mean ± SD for *ALD3* mRNAs. *$p < 0.05$, **$p < 0.01$.

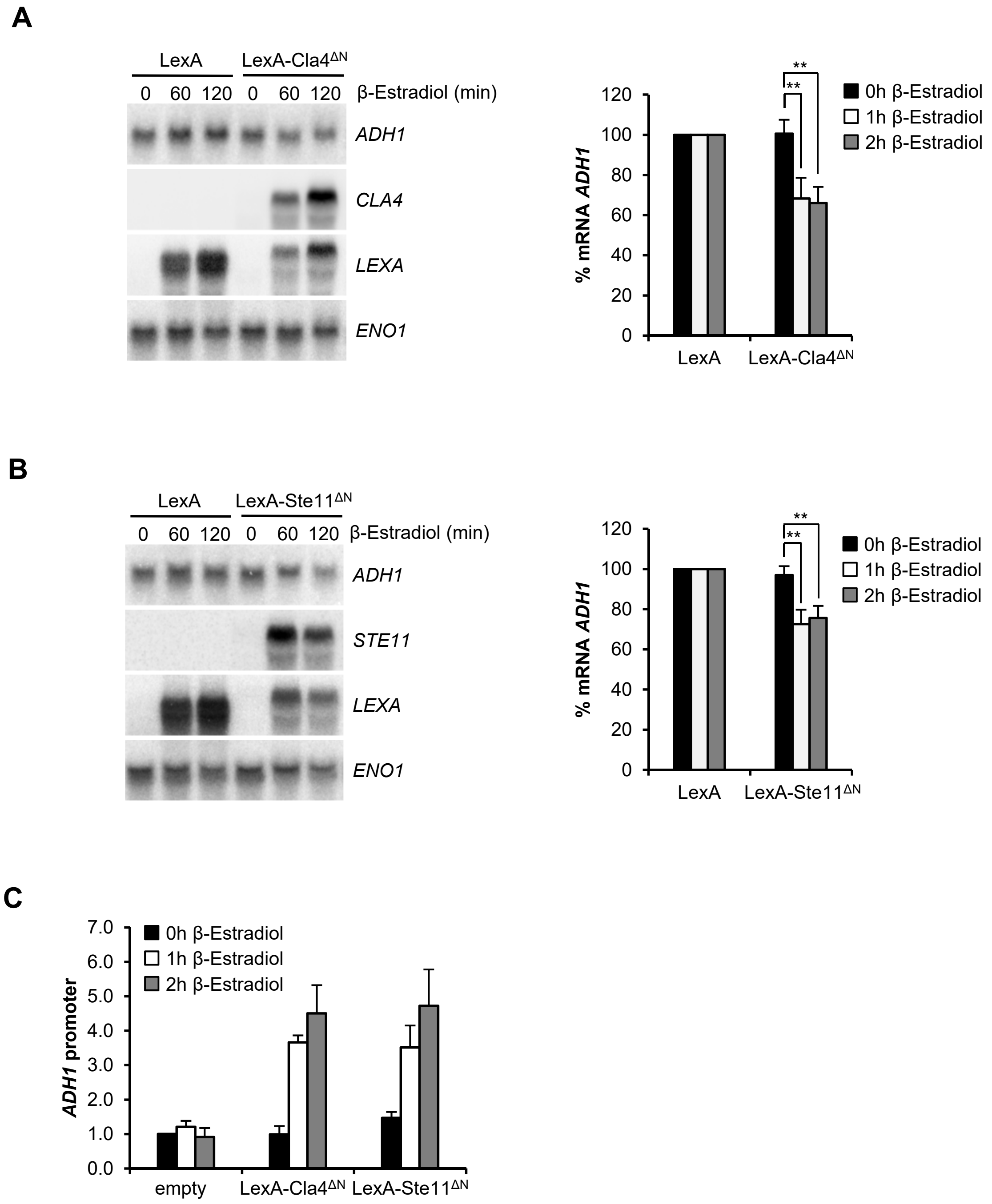

Figure S7. Tethering the hyperactive kinases $Cla4^{\Delta N}$ (A) and $Ste11^{\Delta N}$ (B) down-regulates transcription of *ADH1*. The expression of protein LexA alone (LexA) or fused to kinases $Cla4^{\Delta N}$ (LexA-$Cla4^{\Delta N}$) or $Ste11^{\Delta N}$ (LexA-$Ste11^{\Delta N}$) was induced with β-estradiol for the indicated times in strains harboring the operons of LexA at -400 of *ADH1* gene. Total mRNA was analyzed by northern blot for *ADH1, LEXA*, *CLA4, STE11* and *ENO1* as loading control. A representative figure (left panel) and mRNA quantification of *ADH1* expression of at least three experiments (right panel) is shown for $Cla4^{\Delta N}$ **(A)** and $Ste11^{\Delta N}$ **(B)** tethering. mRNA expression is expressed in percentage as the ratio of *ADH1* normalized by *ENO1* and taking the value of maximum gene expression of the LexA empty strain per time point as 100% reference. Data are reported as mean ± SD. $*p < 0.05$, $**p < 0.01$. **(C)** Binding of LexA-HA fused $Cla4^{\Delta N}$ and $Ste11^{\Delta N}$ was analyzed by ChIP to LexA operons at -400 bp of *ADH1* gene. Real-time PCR results are plotted as the fold induction relative to an untagged strain normalized to a telomere internal control.

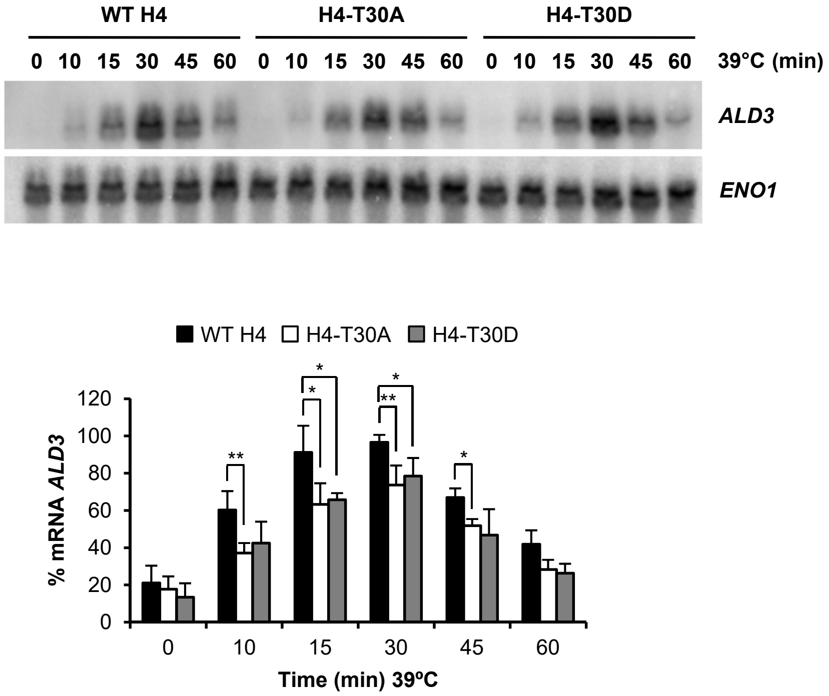

Figure S8. H4-T30 is required for proper transcriptional response upon heat stress. H4 wild-type and H4-T30 mutant strains were heat stressed (39°C) for the indicated time points. Total mRNA was assayed by northern blot against *ALD3* and *ENO1* (as loading control). RNA quantification is expressed in percentage as the ratio of mRNA levels normalized by *ENO1* and using the value of maximum gene expression of the WT strain as 100% reference. The upper panel shows a representative experiment and the lower panel the data of at least three independent experiments reported as mean ± SD. $*p < 0.05$, $**p < 0.01$.

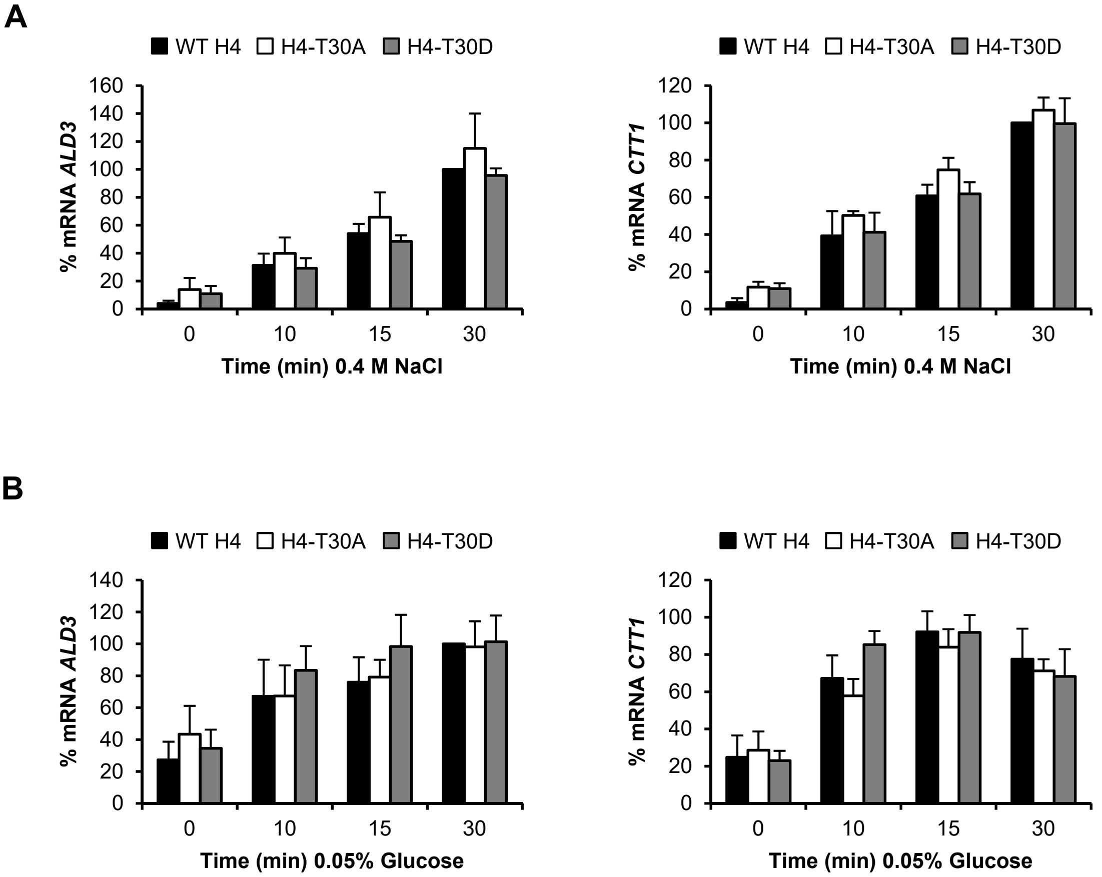

Figure S9. H4-T30 mutant strains does not trigger transcriptional defects upon osmotic and low glucose stress. Wild-type (WT H4), H4-T30A and H4-T30D mutants were subjected to osmotic (0.4 M NaCl) **(A)** or low glucose (0.05%) **(B)** stress for the indicated time points. Total mRNA was analyzed by northern blot for *ALD3*, *CTT1* (stress-responsive genes) and *ENO1* and *RDN18* (loading controls). RNA quantification is expressed in percentage as the ratio of mRNA levels normalized by *ENO1* or *RDN18* and taking the value of maximum gene expression of the WT strain as 100% reference. Data of at least three independent experiments are reported as mean ± SD. *$p < 0.05$, **$p < 0.01$.
